# Supplementary material for: PHOSPHATE STARVATION RESPONSE transcription factors enable arbuscular mycorrhiza symbiosis
Source: Nat Commun. 2022 Jan 25;13:477. doi: 10.1038/s41467-022-27976-8 (PMC8789775; doi:10.1038/s41467-022-27976-8)
Supplement: Supplementary file 1 — Supplementary Information [file 41467_2022_27976_MOESM1_ESM.pdf]

PHOSPHATE STARVATION RESPONSE transcription factors enable arbuscular mycorrhiza symbiosis

Das *et al.*

This PDF file includes:
Supplementary Figures 1 to 25
Supplementary Tables 1 to 5
Supplementary References

Other Supplementary Materials for this manuscript include the following:
Supplementary Data 1 to 10

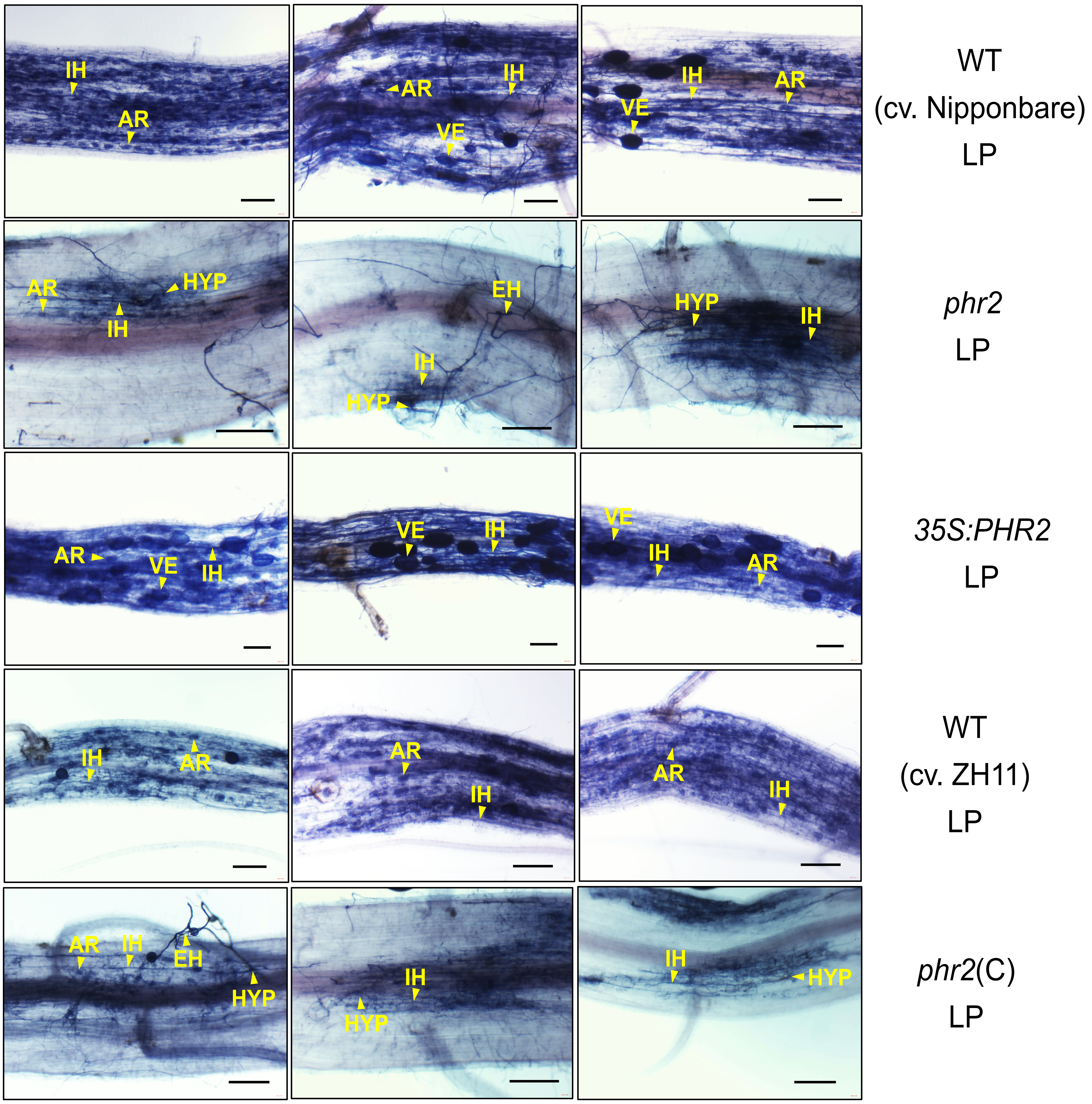

Supplementary Figure 1. Colonization of WT, *phr2* and *35S:PHR2*. Brightfield images of roots stained with acid-ink to visualize colonization of wild type (cv. Nipponbare), *phr2* and *35S:PHR2* and wild-type (cv. ZH11) and *phr2*(C) roots by *Rhizophagus irregularis* at 7 wpi when grown at LP (25 μM P_i) in quartz sand. Scale bars, 200 μm. Abbreviations: EH, extraradical hypha; HYP: hyphopodium; IH, intraradical hypha; AR, arbuscule, VE, vesicle. The phenotype was observed in 11 (6 + 5) independent plants in two independent experiments.

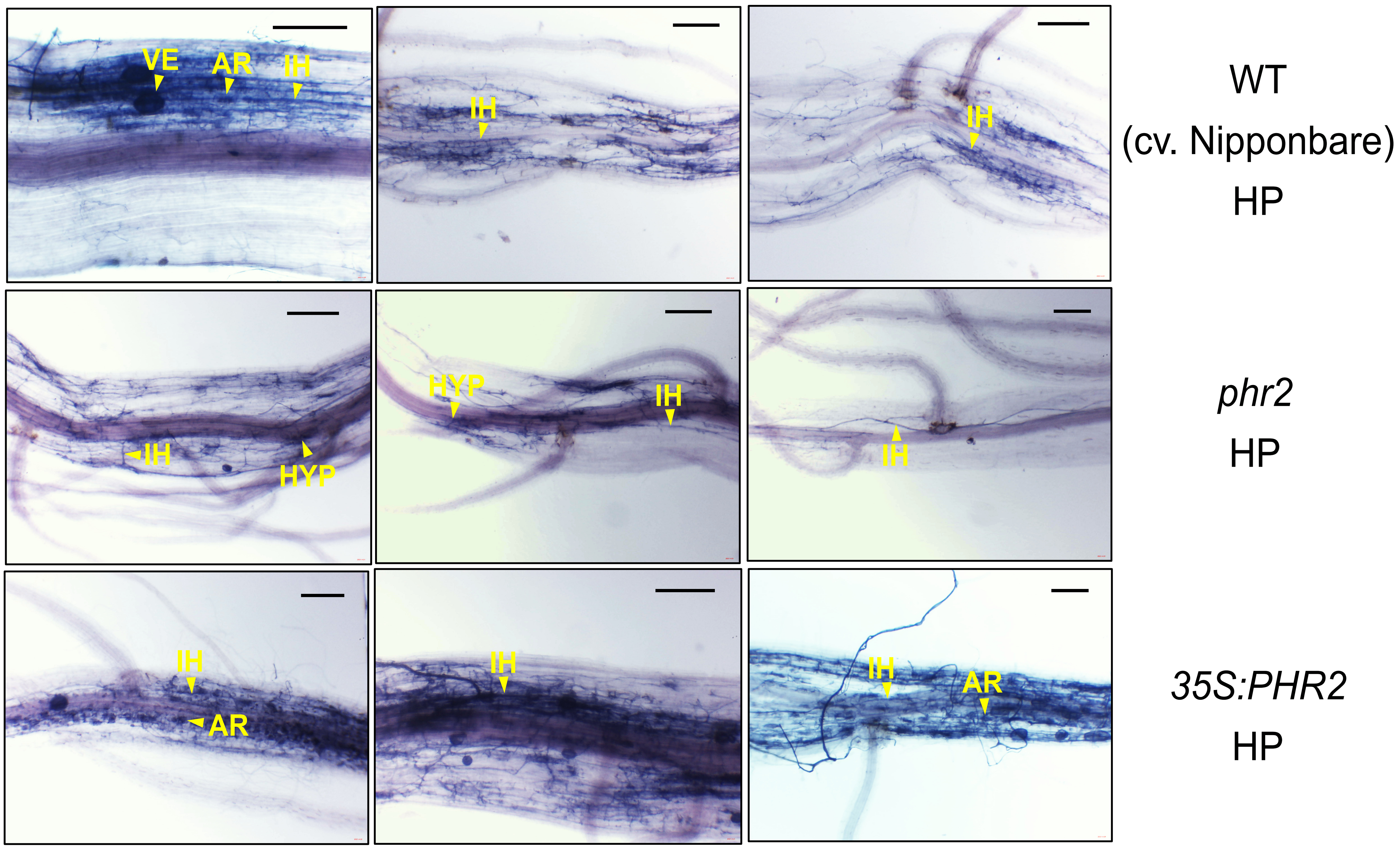

Supplementary Figure 2. Colonization of WT, *phr2* and *35S:PHR2* at high phosphate. Brightfield images of roots stained with acid-ink to visualize colonization of wild type (cv. Nipponbare), *phr2* and *35S:PHR2* roots by *Rhizophagus irregularis* at 7 wpi and grown at HP (500 µm P_i) in quartz sand. Scale bars, 200 µm. Abbreviations: EH, extraradical hypha; HYP: hyphopodium; IH, intraradical hypha; AR, arbuscule, VE, vesicle. The phenotype was observed in 11 (6 + 5) independent plants in two independent experiments.

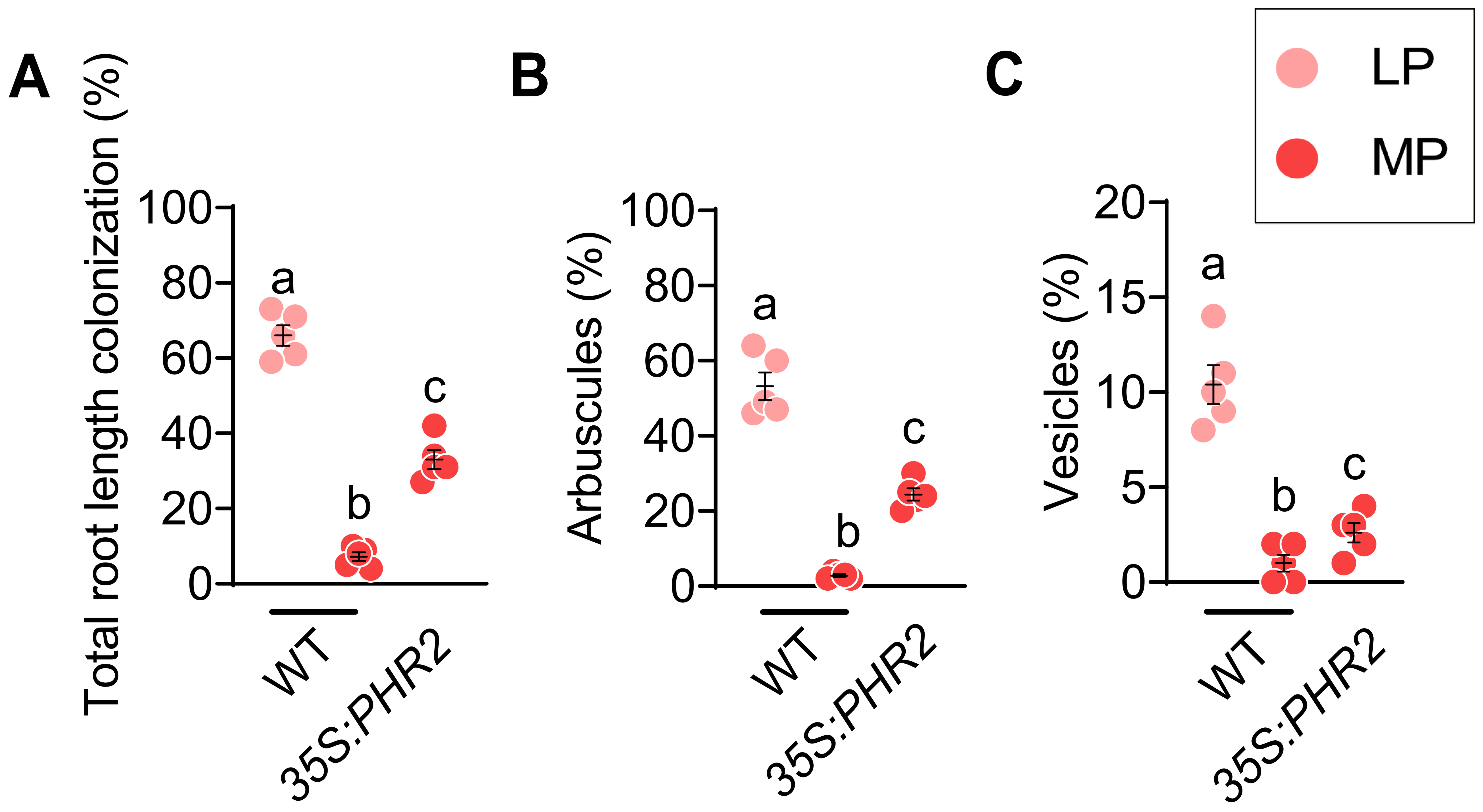

Supplementary Figure 3. Effect of PHR2 overexpression on root colonization by AM fungi at medium phosphate. Effect of low (LP, 25 µM P_i) and medium (MP, 200 µM P_i) phosphate conditions on total root colonization (**A**), arbuscules (**B**) and vesicles (**C**) at 7 wpi. Statistics: Individual data-points and mean ± SE are shown. N=5 independent root systems; Brown-Forsythe and Welch's One-Way ANOVA test with Games-Howell's multiple comparisons test. Different letters indicate statistical differences.

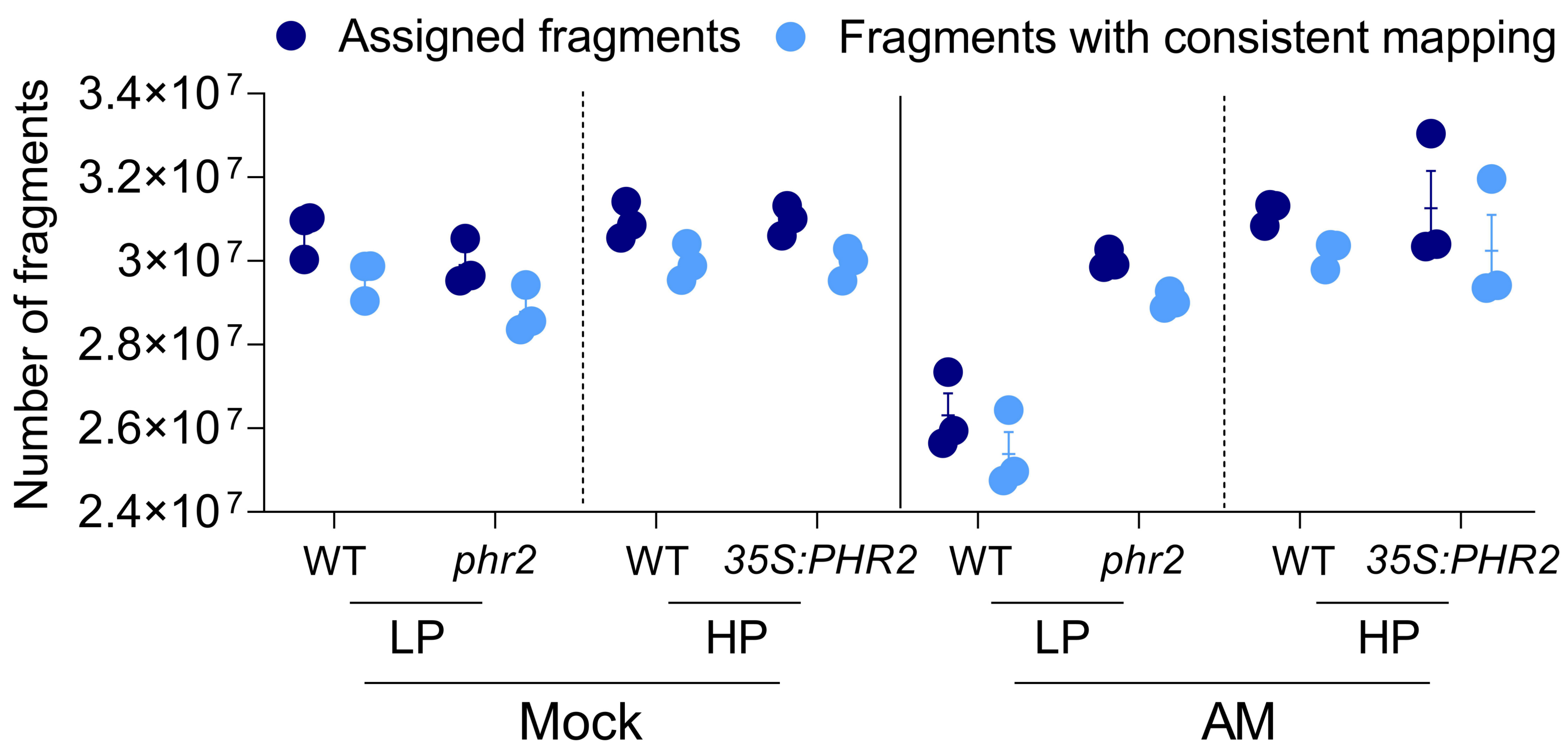

Supplementary Figure 4. RNA-seq fragment mapping. Number of assigned fragments in RNA-Seq along with fragments consistently mapped to the transcriptome reference, N=3 independent root systems. Individual data-points and mean ± SE are shown. Lower number of fragments in AM-inoculated wild-type roots grown at LP results from the fact that these sample also contain reads from *R. irregularis* (≈10% of total fragments).

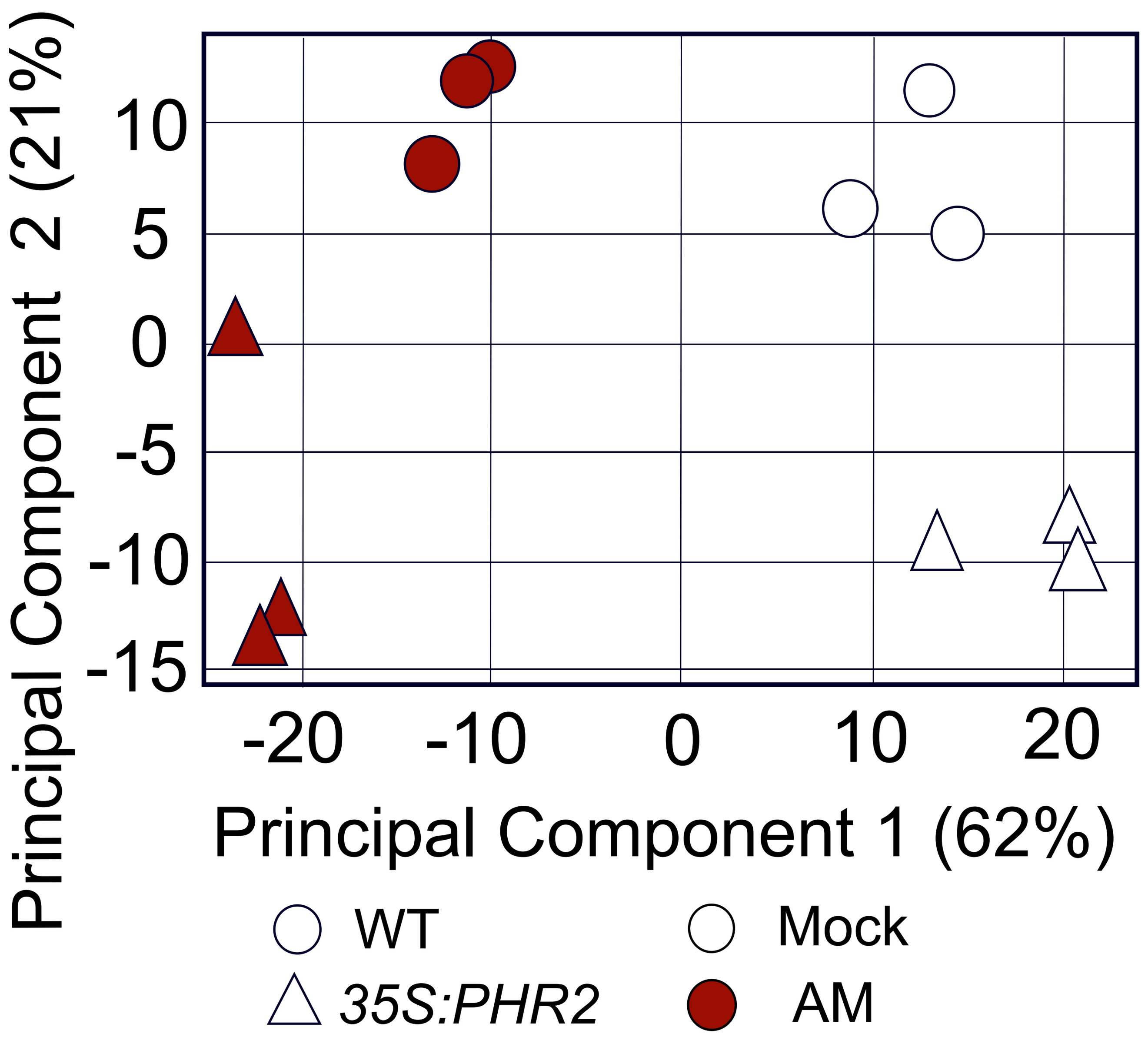

Supplementary Figure 5. PCA plot for high phosphate samples in RNASeq. PCA plot for the RNA-Seq based transcriptome of mock and AMF-inoculated wild-type and *35S:PHR2* roots grown at HP conditions.

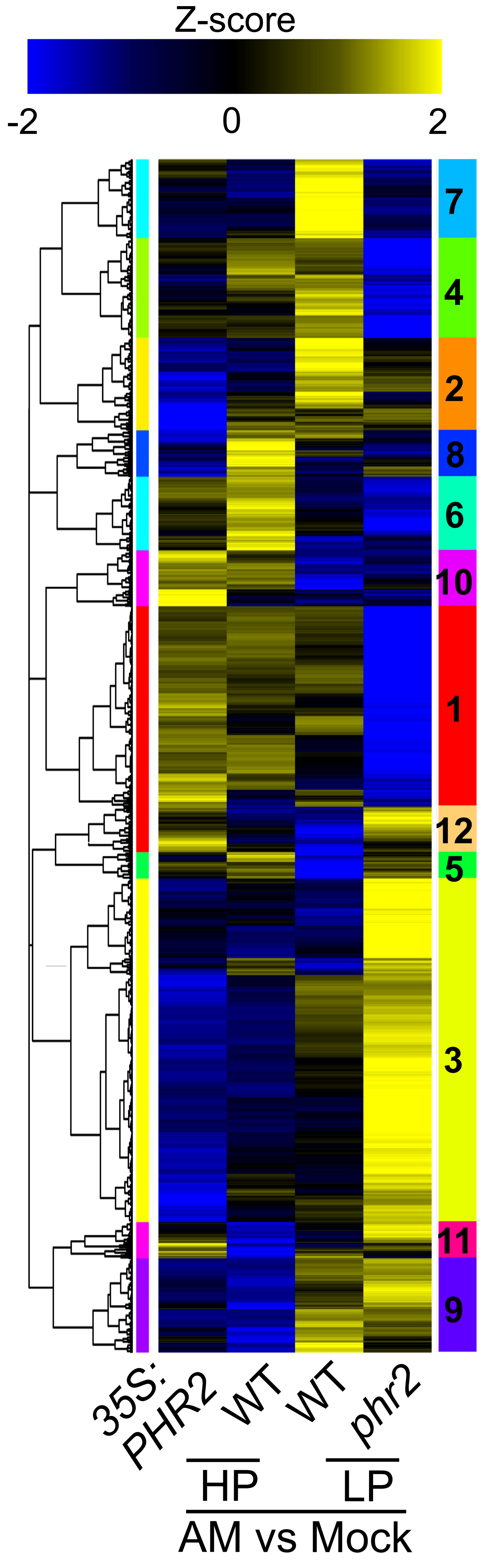

Cluster 7 genes with positive Z-scores in *35S:PHR2* HP and WT LP samples but negative Z-scores in *phr2* LP and WT HP samples

Term	Description	neglogP
GO:0055114	oxidation-reduction process	4.44
GO:0005975	carbohydrate metabolic process	3.94
GO:0006629	lipid metabolic process	2.79
GO:0006633	fatty acid biosynthetic process	2.47
GO:0016114	terpenoid biosynthetic process	2.12
GO:0006952	defense response	1.98
GO:0008219	cell death	1.90
GO:0009239	enterobactin biosynthetic process	1.86
GO:0006915	apoptotic process	1.85
GO:0008610	lipid biosynthetic process	1.83
GO:0009607	response to biotic stimulus	1.82
GO:0055085	transmembrane transport	1.68
GO:0008299	isoprenoid biosynthetic process	1.65
GO:0006631	fatty acid metabolic process	1.31
KEGG:osa00900	Terpenoid backbone biosynthesis	1.29

Cluster 1 DEGs with positive Z-scores in *35S:PHR2* HP and WT LP samples but negative Z-scores in *phr2* LP and WT HP samples

Term	Description	neglogP
GO:0016310	phosphorylation	5.35
GO:0009875	pollen-pistil interaction	4.96
GO:0006468	protein phosphorylation	4.14
GO:0048544	recognition of pollen	3.56
GO:0019748	secondary metabolic process	3.11
GO:0006952	defense response	3.08
GO:0055114	oxidation-reduction process	3.05
GO:0055085	transmembrane transport	3.04
GO:0009607	response to biotic stimulus	2.91
GO:0006636	unsaturated fatty acid biosynthetic process	2.23
GO:0006629	lipid metabolic process	2.16
GO:0046686	response to cadmium ion	2.00
GO:0019288	isopentenyl diphosphate biosynthetic process, methylerythritol 4-phosphate pathway	2.00
GO:0006091	generation of precursor metabolites and energy	1.94
GO:0010218	response to far red light	1.92
GO:0006811	ion transport	1.88
GO:0009744	response to sucrose	1.68
GO:0006865	amino acid transport	1.68
GO:0006813	potassium ion transport	1.62
GO:0016117	carotenoid biosynthetic process	1.51
GO:0019344	cysteine biosynthetic process	1.48
GO:0080167	response to karrikin	1.47
GO:0015995	chlorophyll biosynthetic process	1.45
GO:0006520	cellular amino acid metabolic process	1.39
GO:0003333	amino acid transmembrane transport	1.38
GO:0006655	phosphatidylglycerol biosynthetic process	1.36
KEGG:osa00904	Diterpenoid biosynthesis	2.23
KEGG:osa00400	Phenylalanine, tyrosine and tryptophan biosynthesis	1.77

Supplementary Figure 6. Hierarchical clustering of combined DEGs. Hierarchical clustering of combined DEGs (AM vs Mock samples, log_2(Fold-change), lfc) from roots of *phr2* and *35S:PHR2* in LP and HP respectively and wild type at both P_i conditions. Z-scores represent scaled lfc. Colored bars on the left side of heatmap depict individual clusters (based on the dendrogram). Gene ontology (GO) enrichment analysis for selected DEGs in cluster-1 and -7, which showed positive Z-scores for *35S:PHR2* HP and WT LP samples and negative Z-scores for *phr2* LP and WT HP samples. GO term enrichment indicates functional categories important for AM symbiosis such as fatty acid, lipid and carotenoid metabolism, response to biotic stimulus and response to karrikin.

A

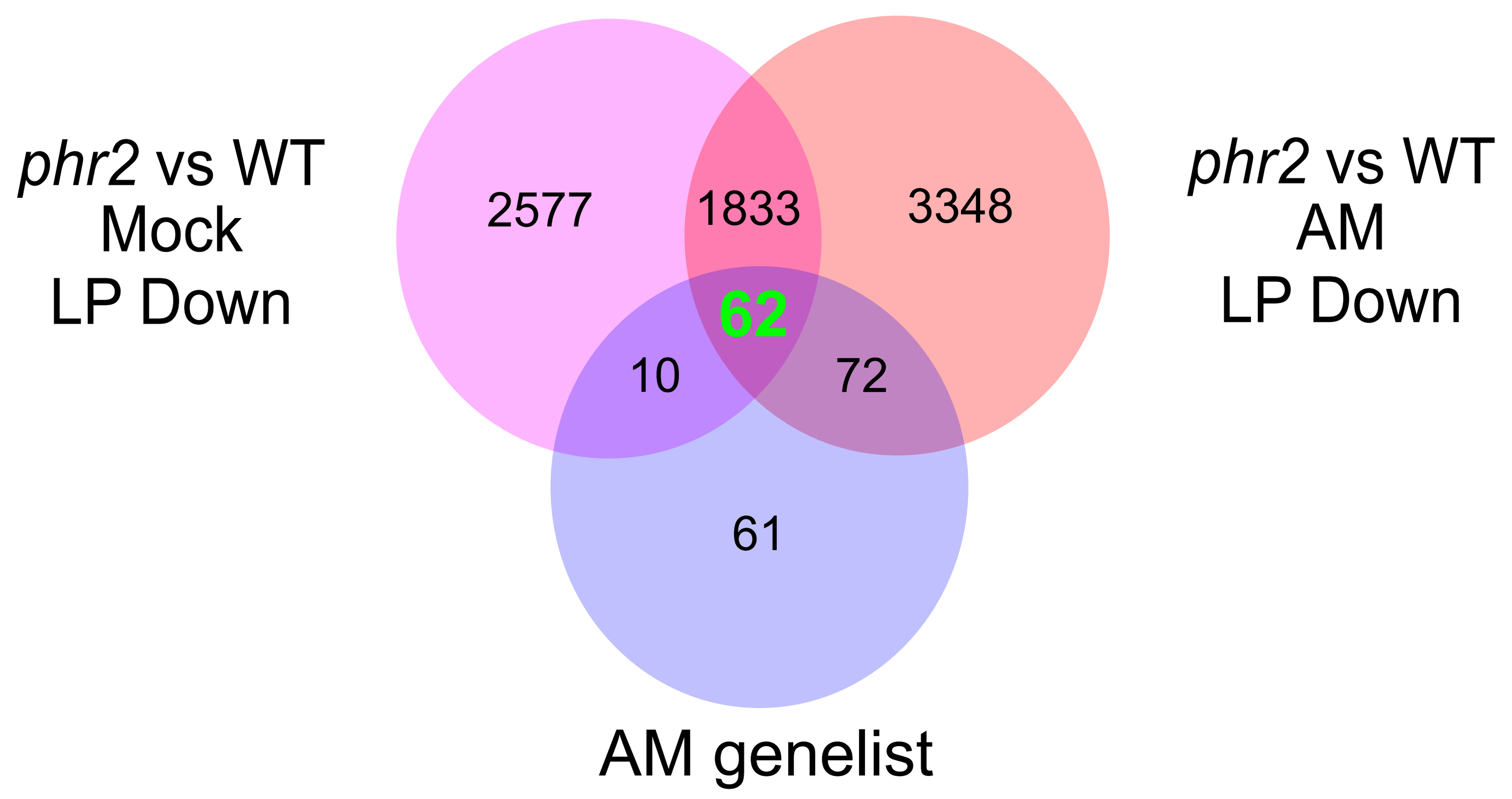

B

Locus ID (62 Genes)	Gene description
**LOC_Os01g65000**	**AMT3;1, ammonium transporter protein**
**LOC_Os03g45290**	**Ankyrin repeat domain-containing protein (Medtr6g027840 VAPYRIN)**
**LOC_Os01g54270**	**D10, CCD8B**
**LOC_Os01g38580**	**D10-like, CCD8A**
**LOC_Os04g46470**	**D17, CCD7, carotenoid cleavage dioxygenase 7**
**LOC_Os11g01050**	**EXO70 exocyst complex subunit domain containing protein**
**LOC_Os03g29480**	**NSP1, GRAS transcription factor, nodulation-signaling pathway 1 protein**
**LOC_Os03g15680**	**NSP2, nodulation-signaling pathway 2 protein**
**LOC_Os05g41090**	**OsDMI3, OsCCaMK, calcium/calmodulin depedent protein kinases**
**LOC_Os06g02520**	**OsIPD3, OsCYCLOPS, interacting protein of DMI3**
**LOC_Os03g13080**	**OsLysM-RLK2,OsLYK5, MYR1/LYK2/RLK2/NFR5**
**LOC_Os06g44430**	**Protein kinase (Medtr4g129010 KIN2)**
**LOC_Os01g46860**	**PT11, inorganic phosphate transporter**
**LOC_Os04g10800**	**PT13, inorganic phosphate transporter**
**LOC_Os09g23640**	**STR1, ABC-2 type transporter domain containing protein**
**LOC_Os07g38070**	**SYMRK, protein kinase, putative, expressed (Lj2g3v1467920 LjSymRK)**
**LOC_Os09g15240**	**Zaxinone Synthase (ZAS), carotenoid cleavage dioxygenase**
LOC_Os08g05690	ABC transporter, ATP-binding protein, putative
LOC_Os05g50300	AMP1, AMP-binding enzyme, putative
LOC_Os04g39780	AMP-binding enzyme family protein
LOC_Os01g44950	AMP-binding enzyme, putative
LOC_Os07g38750	AP2 domain containing protein
LOC_Os12g39330	AP2 domain containing protein
LOC_Os02g48820	BCP, plastocyanin-like domain containing protein, putative, expressed
LOC_Os06g47130	C2domain containing protein
LOC_Os07g23450	C2H2 zinc finger protein
LOC_Os08g28240	carotenoid cleavage dioxygenase, putative
LOC_Os04g58680	CBF1/2, core histone H2A,H2B,H3,H4, putative
LOC_Os06g20120	CND41, chloroplast nucleoid DNA binding protein, putative, expressed
LOC_Os09g39530	Cupin-domain contaning protein
LOC_Os07g33620	cytochrome P450 domain containing protein
LOC_Os01g50520	cytochrome P450 monooxygenase CYP711A12, putative, expressed
LOC_Os01g50590	cytochrome P450, putative, expressed
LOC_Os05g51240	D14L2a, Hydrolase, alpha/beta fold family domain containing protein
LOC_Os10g42400	DNA polymerase III, clamp loader complex, gamma/delta/delta subunit
LOC_Os01g46390	DUF538 domain containing protein, putative
LOC_Os05g49790	DUF538 domain containing protein, putative
LOC_Os08g42990	expressed protein
LOC_Os11g29630	expressed protein
LOC_Os02g18954	GDSL-like lipase,acylhydrolase, putative
LOC_Os02g44850	GDSL-like lipase,acylhydrolase, putative
LOC_Os04g47390	GDSL-like lipase,acylhydrolase, putative
LOC_Os07g19040	glycosyl hydrolase, putative
LOC_Os08g34258	inhibitor I family protein, putative
LOC_Os08g34249	inhibitor I family protein, putative, expressed
LOC_Os04g39180	KIN6, nodulation receptor kinase precursor, putative
LOC_Os02g20140	KinD, protein kinase domain containing protein
LOC_Os01g57400	lysM domain containing protein, putative
LOC_Os04g40570	MDR1, ABC transporter, ATP-binding protein, putative
LOC_Os05g47500	MDR-like ABC transporter
LOC_Os08g42590	mtN19, putative, expressed
LOC_Os01g52750	OsSub3 - Putative Subtilisin homologue
LOC_Os04g04750	peroxidase precursor, putative, expressed
LOC_Os03g57740	plastocyanin-like domain containing protein, putative
LOC_Os02g57700	protein kinase, putative, expressed
LOC_Os01g72710	putative RETICULATA-RELATED protein of unknown function
LOC_Os09g20970	receptor kinase, putative
LOC_Os03g38600	secretory carrier-associated membrane protein, putative, expressed
LOC_Os08g29510	Taurine catabolism dioxygenase TauD/TfdA domain containing protein
LOC_Os10g18510	UDP-glucoronosyl and UDP-glucosyl transferase domain containing protein
LOC_Os05g50680	WRKY83
LOC_Os01g53130	zinc finger, C3HC4 type domain containing protein, expressed

Supplementary Figure 7. Overlap of genes with decreased transcript levels in *phr2* in Mock or AM roots with AM genelist. (**A**) Venn diagram of DEGs downregulated in *phr2* vs WT Mock and AM roots at LP and AM genelist. (**B**) Genes common to all the three sets. Genes highlighted in red have been previously genetically shown to be required for AM development or function.

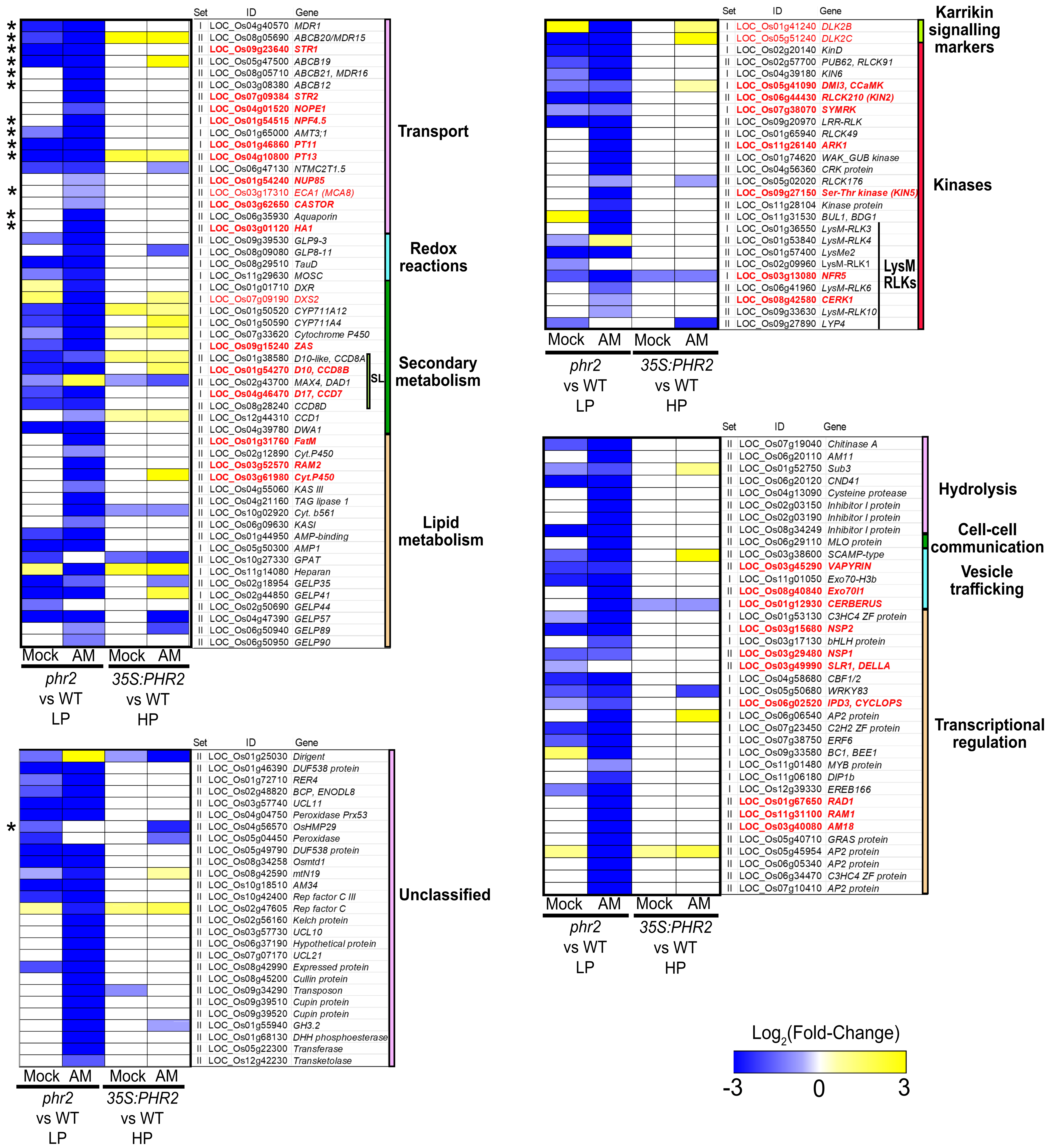

Supplementary Figure 8. Expression of genes required for or induced during AM depends on PHR2. Heatmaps for $\log_2$(Fold-change) of AM genes from set I and II (Figure 2G) for the comparisons *phr2* vs wild type at LP (25 µM) and *35S:PHR2* vs wild type at HP (500 µM). Colored bars on the right indicate functional categories to which the genes belong. Genes with genetically confirmed functions in AM symbiosis are indicated in red (bold for mutants, regular font for *RNAi* lines). 144 out of 205 genes (70%) in the AM genelist had reduced expression in *phr2* vs WT AM and/or Mock LP samples. Asterisks indicate orthologs of *Lotus japonicus* genes involved in transport and associated with P1BS elements in Supplementary Table 2[1]. Out of 48 total genes in this table, only 31 could be retrieved from the *Lotus japonicus* genome assembly build 1.0 (http://www.kazusa.or.jp/lotus/release1/) and 14 out of these have reduced expression in rice *phr2* vs WT).

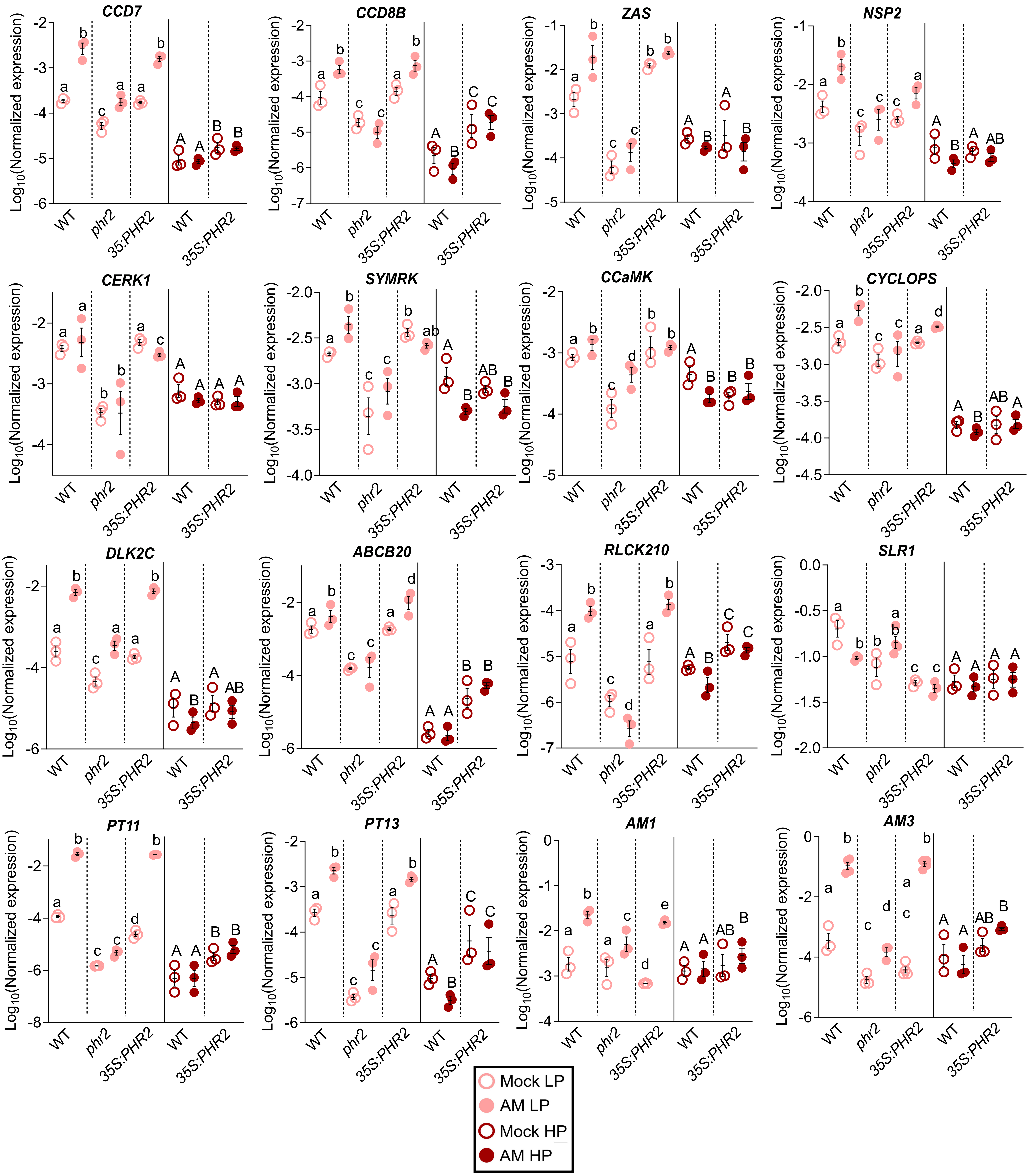

Supplementary Figure 9. RT-qPCR-based transcript accumulation of selected DEGs recapitulates the RNA-Seq results. Relative transcript accumulation in mock inoculated (Mock) and *R. irregularis* colonized (AM) roots of the indicated genotypes grown in quartz sand and fertilized with LP (25 µM P_i) or HP (500 µM P_i). Expression values of indicated genes were normalized to the geometric mean of the expression of two housekeeping genes, *UBIQUITIN* and *CYCLOPHILIN.* Letters indicate statistical differences between genotypes and treatments for each phosphate level separately. Statistics: Individual data-points and mean ± SE are shown. N=3 biologically independent samples. Brown-Forsythe and Welch's One-Way ANOVA test with Games-Howell's multiple comparisons test was carried out between genotypes at each phosphate level separately. Different letters indicate statistical differences.

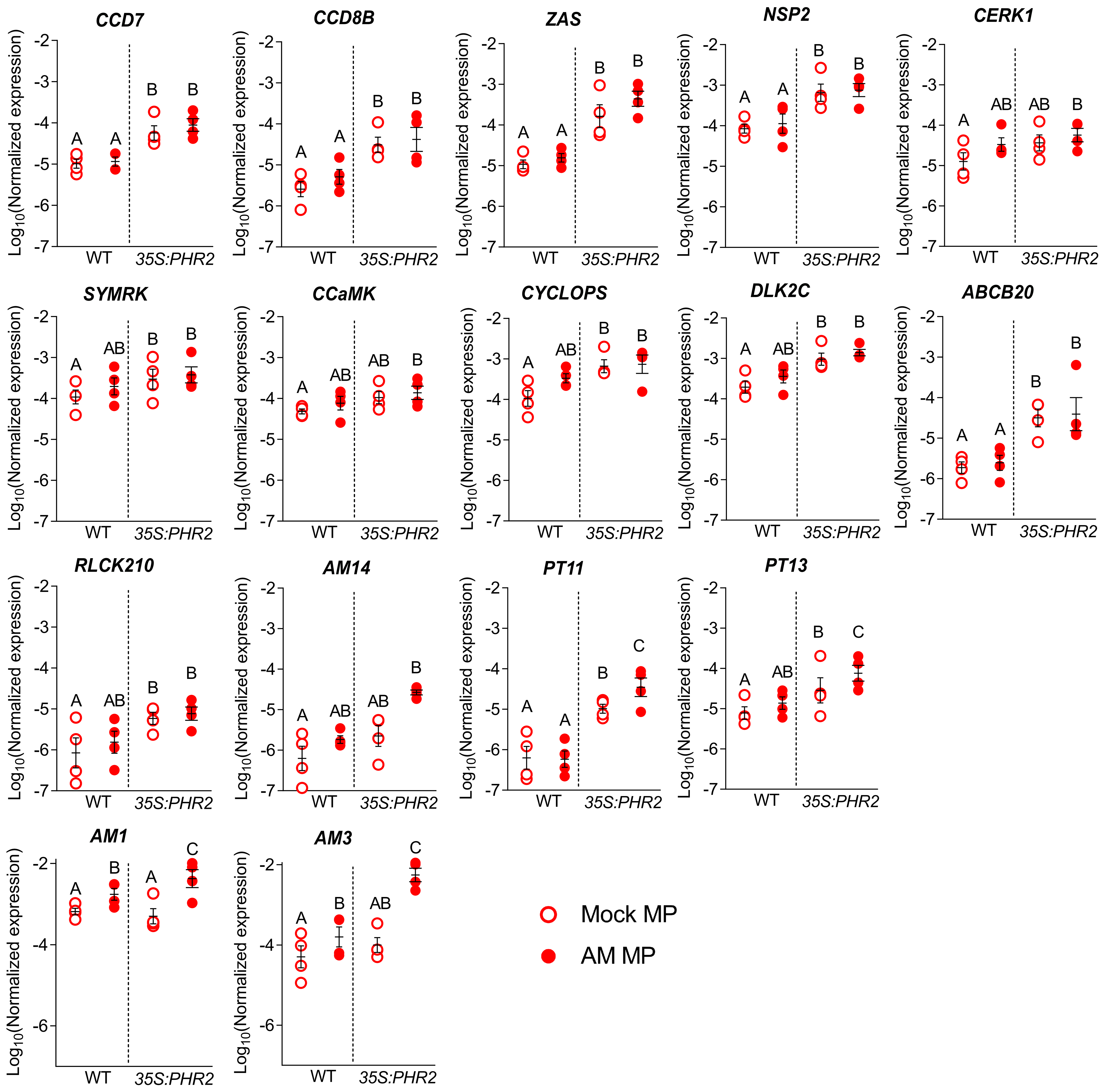

Supplementary Figure 10. RT-qPCR-based transcript accumulation of selected DEGs at medium phosphate. Relative transcript accumulation in mock inoculated (Mock) and *R.irregularis* colonized (AM) roots of the indicated genotypes grown in the same experiment as Fig. S3 in quartz sand and fertilized with MP is shown. Expression values of indicated genes were normalized to the geometric mean of the expression of two housekeeping genes, *UBIQUITIN* and *CYCLOPHILIN.* Letters indicate statistical differences between genotypes and treatments within each phosphate level. Statistics: Individual data-points and mean ± SE are shown. N=3-4 biologically independent samples. Brown-Forsythe and Welch's One-Way ANOVA test with Games-Howell's multiple comparisons test was carried out. Different letters indicate statistical differences.

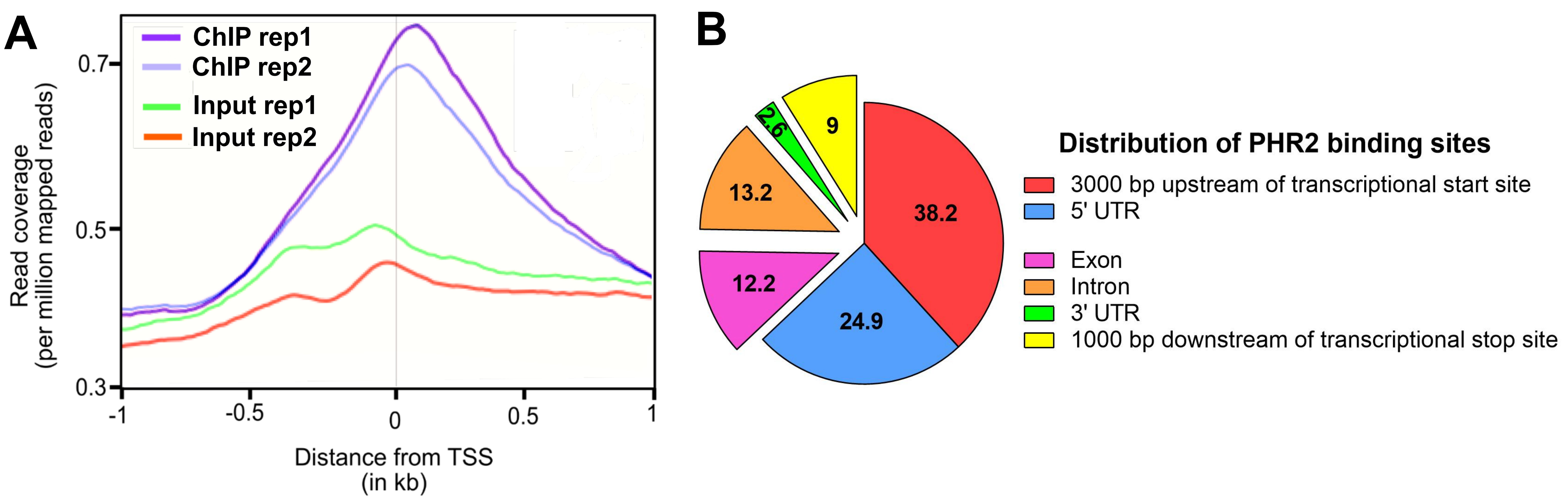

Supplementary Figure 11. ChIP-Seq binding peaks of PHR2-FLAG are enriched near the transcriptional start site. (**A**) Read coverage plot for the two biological replicates from ChIP-Seq with FLAG tagged PHR2 protein. TSS, transcriptional start site. (**B**) Distribution of PHR2-binding sites in the rice genome. ChIP-Seq read distribution in relation to transcriptional start site (TSS) suggested a slight skew in the distribution of PHR2 binding sites towards 1000 bp downstream of TSS. Correspondingly, PHR2 binding sites are enriched not only in 3000 bp region upstream of TSS (38.2%) but also in the regions downstream of TSS such as 5′ UTR, exon and intron (24.9% + 12.2% + 13.2% = 50.3%).

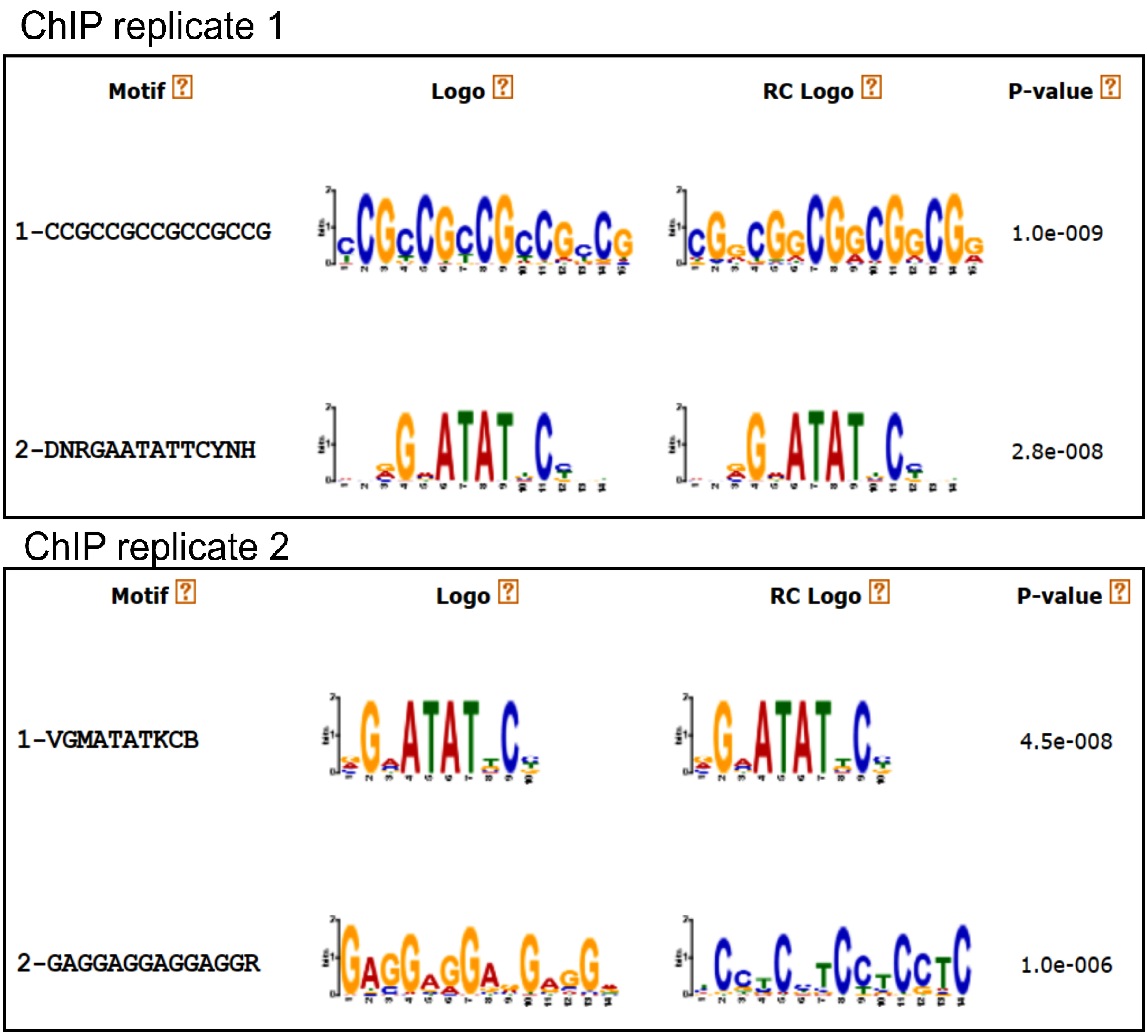

Supplementary Figure 12. Motifs over-represented in DNA sequences with PHR2 binding sites. Analysis was carried out separately for the two biological ChIP-Seq replicates using STREME (https://meme-suite.org/meme/tools/streme).

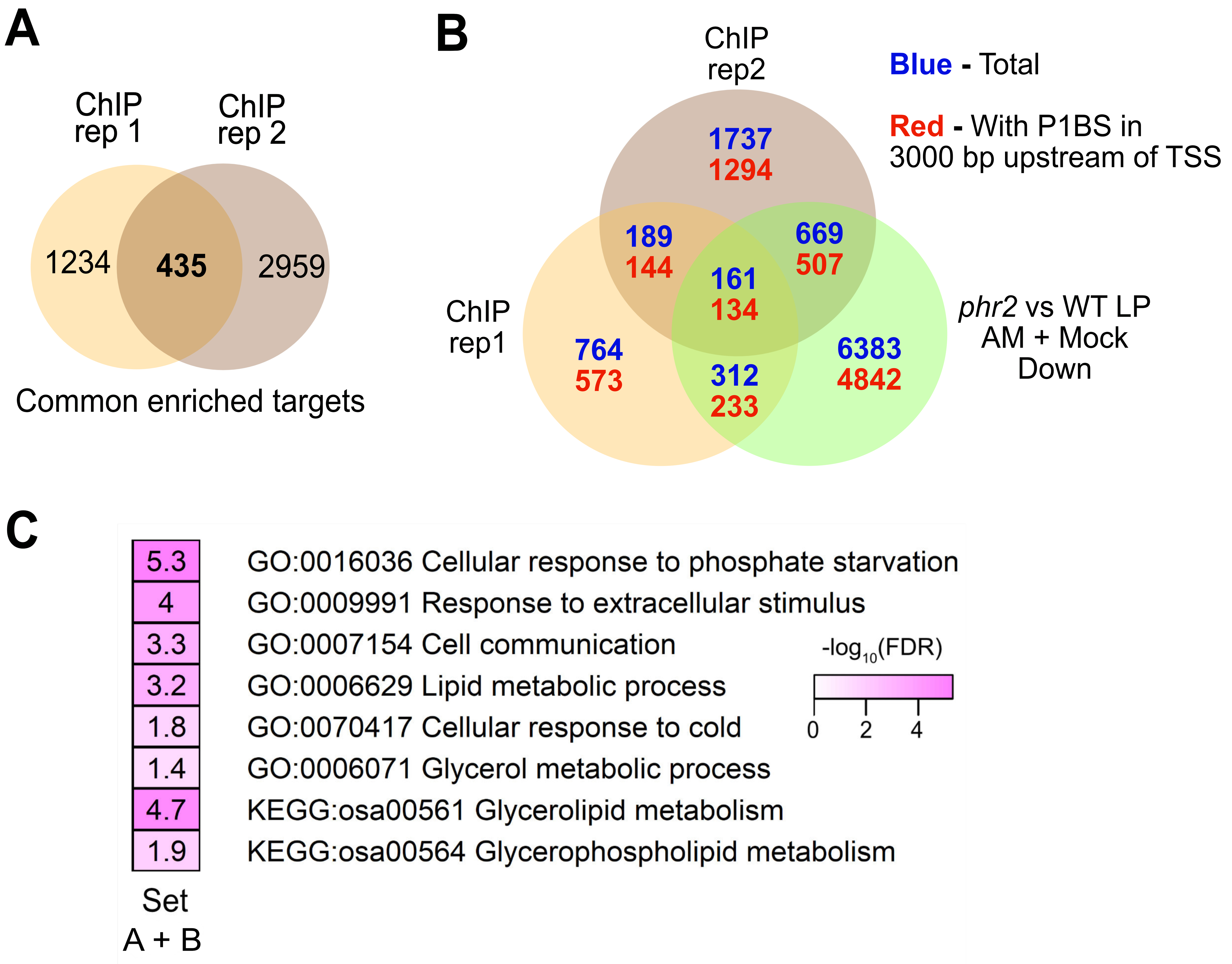

D

**Enriched motifs (Set A + B)**	**E-value**	**Significance**
GAATATGC | GCATATTC	1.00E-05	4.99
GAATATCC | GGATATTC	5.00E-05	4.3

Supplementary Figure 13. Binding site analysis for rice PHR2. (**A**) Venn diagram showing overlap of PHR2 ChIP targets from two independent biological ChIP-Seq replicates. These 435 common PHR2 ChIP targets are genes annotated closest to PHR2-binding sites in both replicates. (**B**) Venn diagram showing overlap of PHR2 targets with DEGs with reduced expression in *phr2* vs WT AM + Mock samples at LP. Blue indicates the total number of genes and red with P1BS or P1BS-like motif in 3000 bp upstream region upstream of transcriptional start site (TSS)). MSU IDs in the three individual gene sets were converted to RAPDB Locus IDs (to facilitate extraction of upstream sequence from RAPDB website, https://rapdb.dna.affrc.go.jp/). This resulted in a smaller number of genes than the original number of MSU ID DEGs. (**C**) GO-term enrichment in category “biological process” for Set A + Set B genes (167 genes out of 435 PHR2 targets which are repressed in AM or Mock root samples of *phr2* vs WT grown at LP as shown in Fig. 3A). Darker colors indicate stronger enrichment of GO-terms. GO-terms include categories involved in phosphate starvation signalling as well as AM. (**D**) Motifs enriched in 1000 bp sequence (upstream of TSS) for Set A + B genes in Fig. 3A.

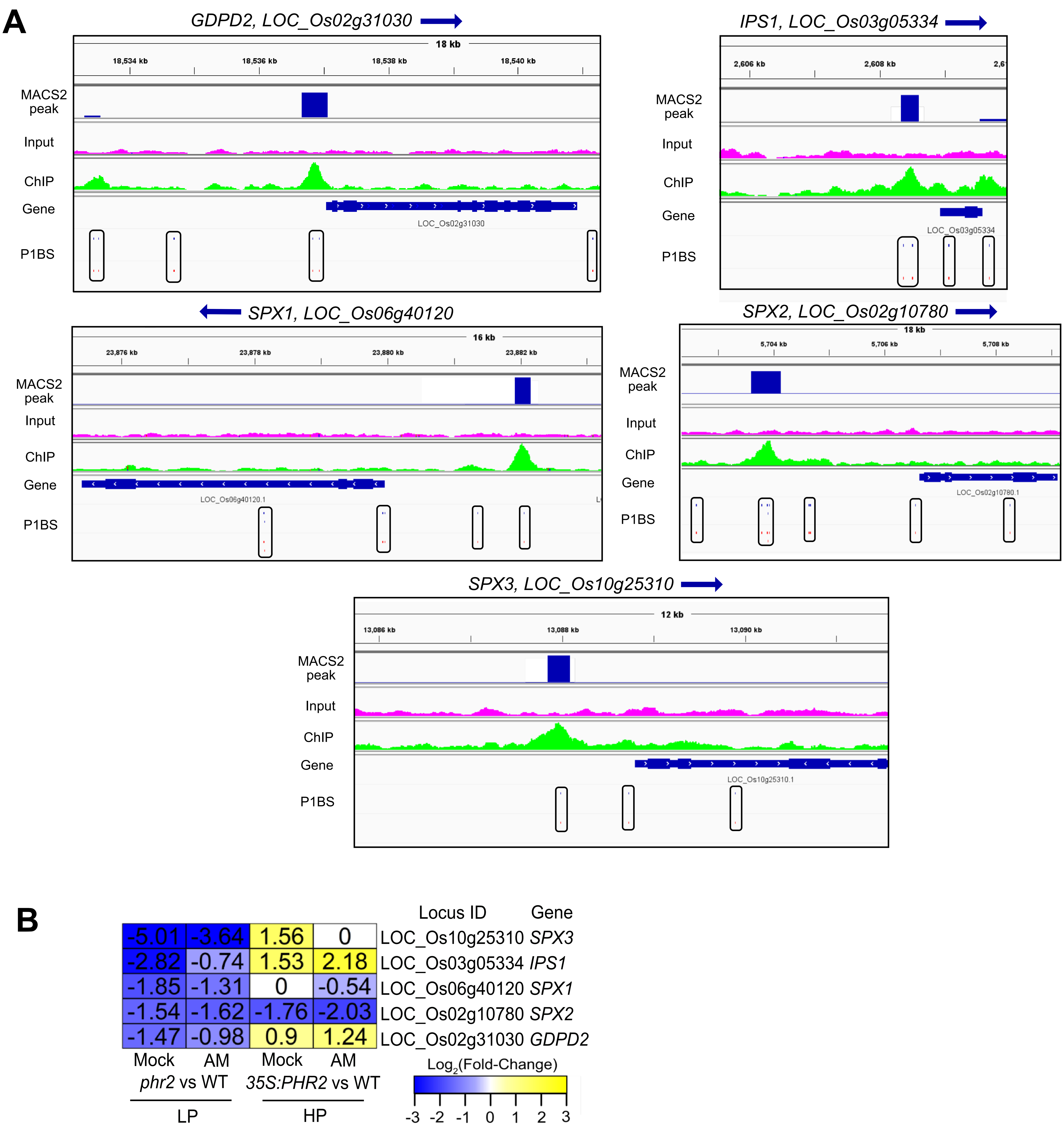

Supplementary Figure 14. IGV browser view of ChIP-Seq peaks adjacent to previously known PHR2 target genes. (**A**) ChIP-Seq peak profiles of genes which have been previously shown to be PHR2 targets. Gene orientation is indicated by the direction of the blue arrow close to the gene name. MACS2 peaks (blue bars) denote PHR2 binding sites corresponding to enrichment of PHR2-FLAG IP (green color) sequencing reads vs Input (pink color) sequencing reads. Positions of P1BS elements along the genomic coordinate are marked by enclosing the motifs in black rectangular boxes. (**B**) RNA-Seq based $\log_2$(Fold-change) of these known PHR2 target genes in for *phr2* vs wild type and *35S:PHR2* vs wild type at LP (25 µM) and HP (500 µM), respectively. The phosphate level at HP (500 µM) maybe high enough to prevent the transcriptional induction of some of these phosphate starvation response genes such as *SPX1* and *2* in the *35S:PHR2* line.

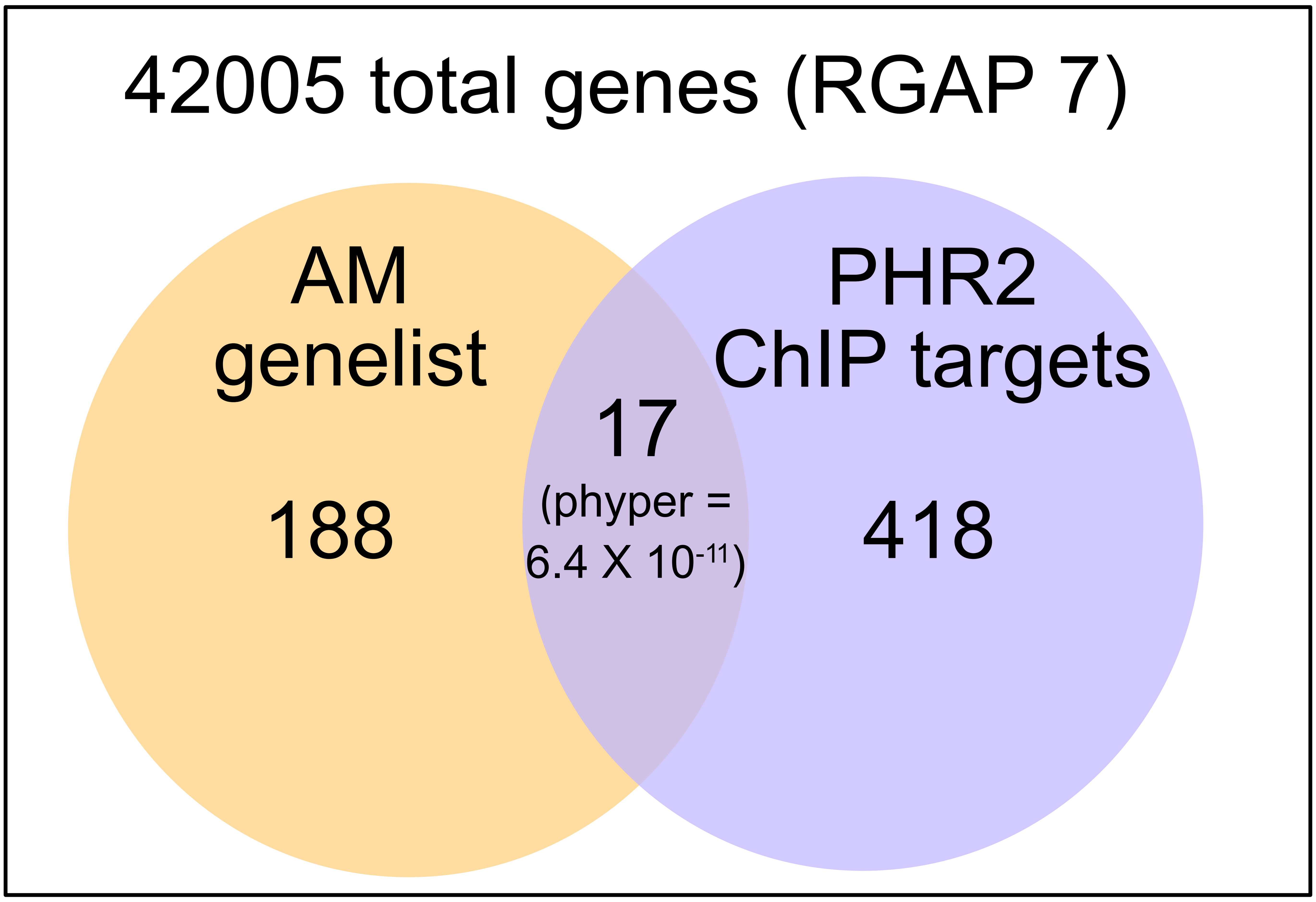

Supplementary Figure 15. Enrichment of PHR2 ChIP targets in AM genelist. Hypergeometric test was used to assess the statistical significance (phyper) of overlap of PHR2 ChIP targets with the AM genelist.

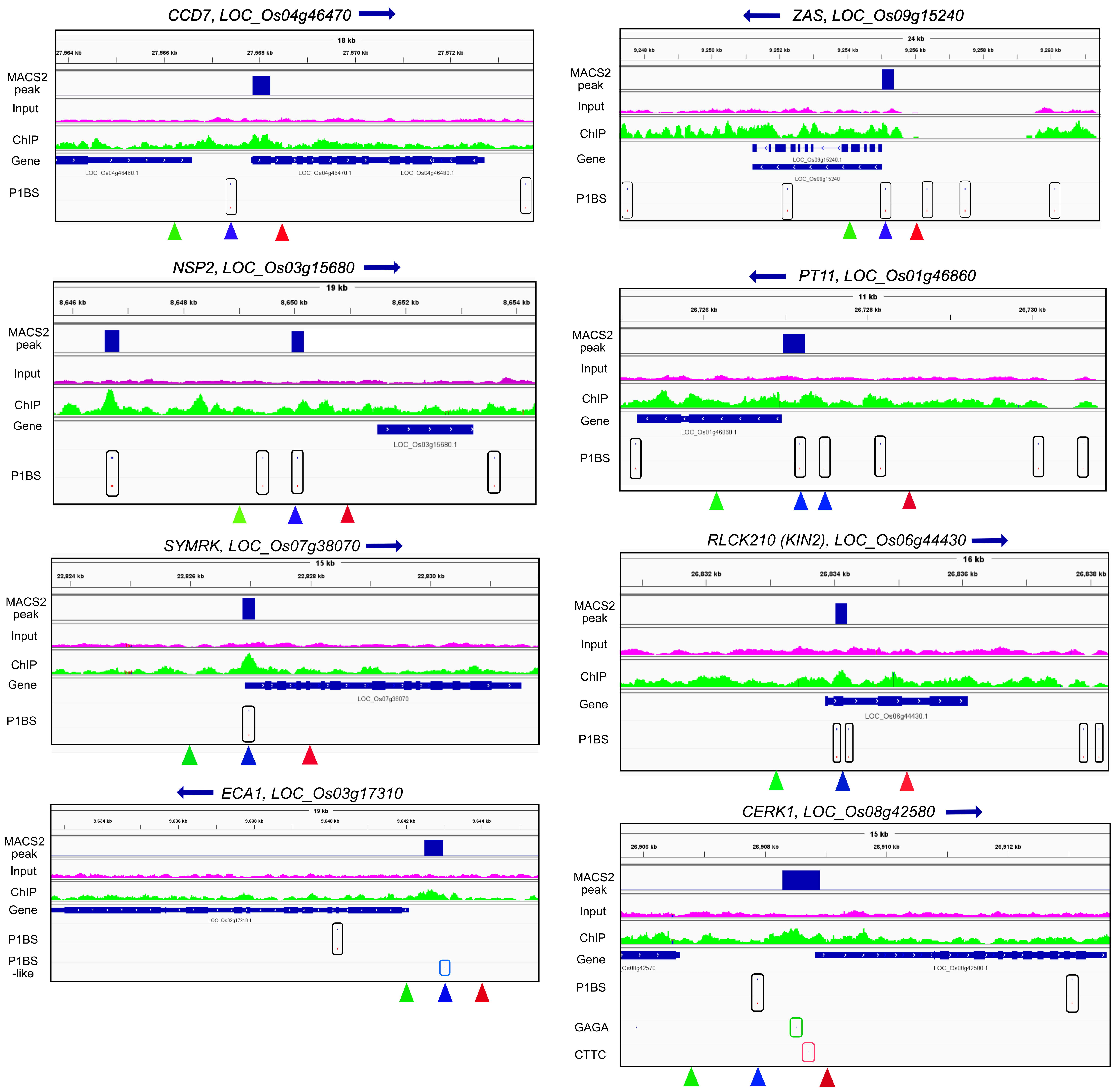

Supplementary Figure 16. IGV browser view of ChIP-Seq peaks adjacent to AM-relevant genes. The selected genes are those depicted in red in Fig. 3C. Gene orientation is indicated by the direction of the blue arrow next to the gene name. MACS2 peaks (blue bars) indicate PHR2 binding sites corresponding to enrichment of PHR2-FLAG IP (green color) sequencing reads vs Input (pink color) sequencing reads. The positions of P1BS elements along the genomic coordinate are marked by enclosing the motifs in black rectangular boxes. In the *ECA1* promoter, the P1BS-like motif is marked by a blue rectangular box. In the *CERK1* promoter, the GAGA and CTTC motifs are marked by enclosing them in green and red rectangular boxes, respectively. Blue triangles indicate the position of ChIP-qPCR (Fig. S15) primers flanking the P1BS motifs, while green and red triangles indicate the position of primers 1000 bp left (5') of P1BS (-1000 bp) and +1000 bp right (3') of P1BS (+1000 bp), respectively. Motifs: P1BS is GNATATNC; P1BS-like is AMATATYC; GAGA is GGAGAGGA; CTTC is TCCTCTTGTTCTTC.

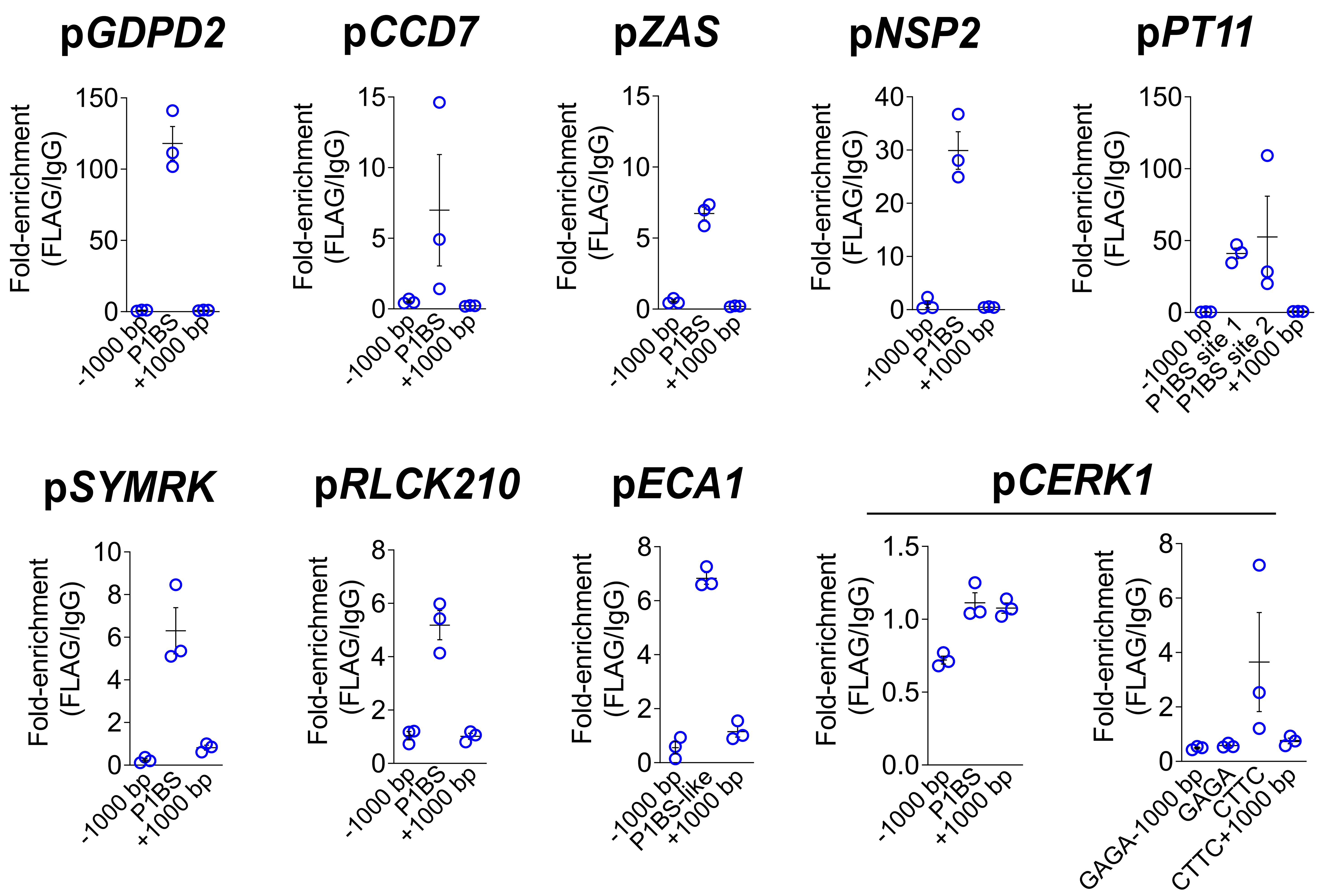

Supplementary Figure 17. Enrichment of PHR2 at P1BS promoter motifs detected by ChIP-qPCR. Primers were designed to amplify regions flanking motifs (P1BS, P1BS-like, GAGA, CTTC), and 1000 bp left (5') of these motifs (-1000 bp) and 1000 bp right (3') of these motifs (+1000 bp). Motifs: P1BS is GNATATNC; P1BS-like is AMATATYC; GAGA is GGAGAGGA; CTTC is TCCTCTTGTTCTTC. Data: Individual data-points and mean ± SE are shown. N=3 biologically independent samples.

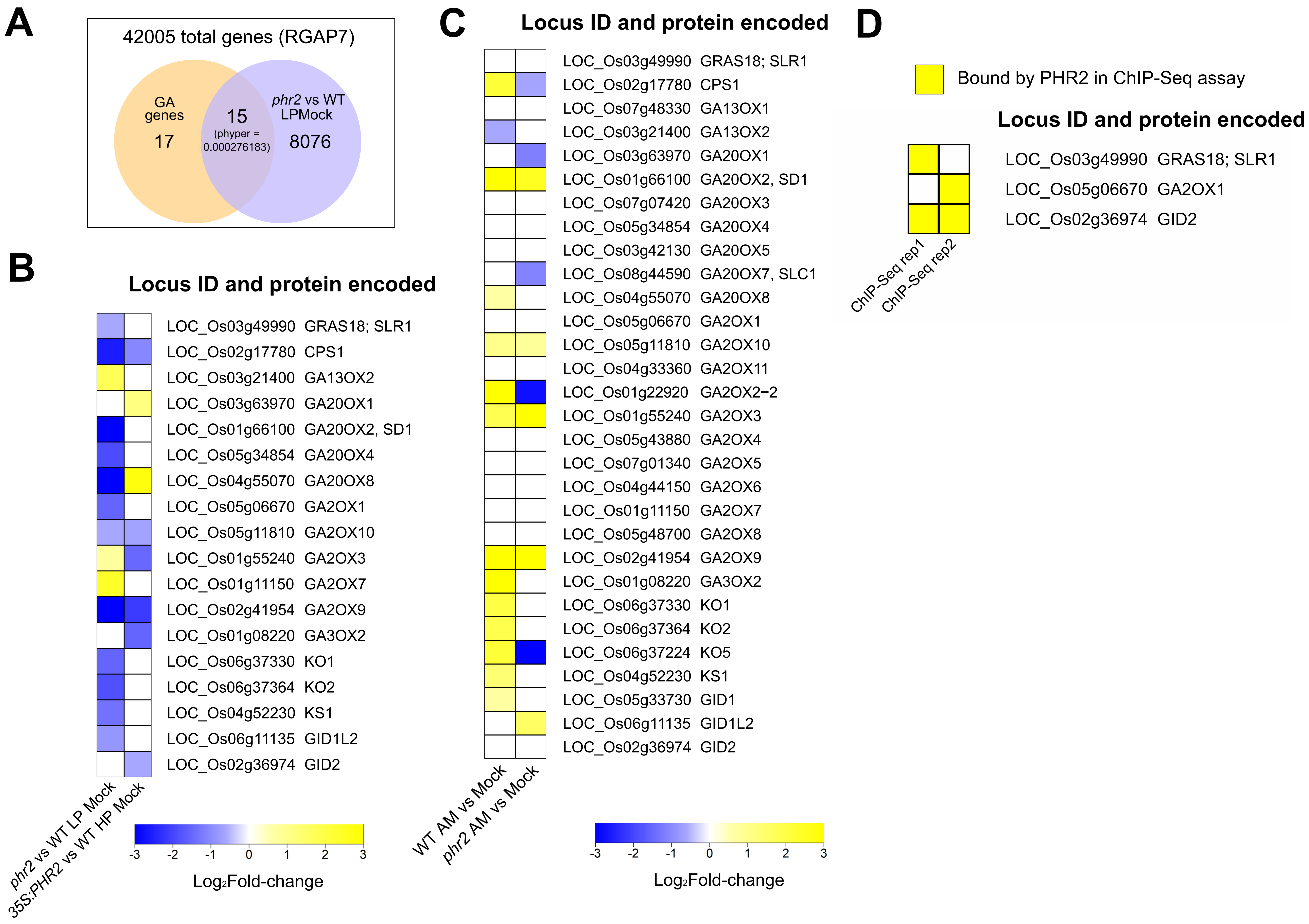

Supplementary Figure 18. Gibberellin-biosynthesis and -signaling related genes in RNASeq and ChIP-Seq. (**A**) Gibberellin (GA)-related genes are enriched in DEGs with reduced expression in non-inoculated *phr2* vs wild type. as shown by a hypergeometric test to assess the statistical significance (phyper). (**B**) Expression of GA-related genes in non-colonized *phr2* vs wild type roots at LP and *35S:PHR2* vs wild type roots at HP. (**C**) Comparison of gene expression for GA-related genes in *phr2* and wild type in AM vs Mock roots. (**D**) GA-related genes directly targeted by PHR2 as determined by ChIP-Seq.

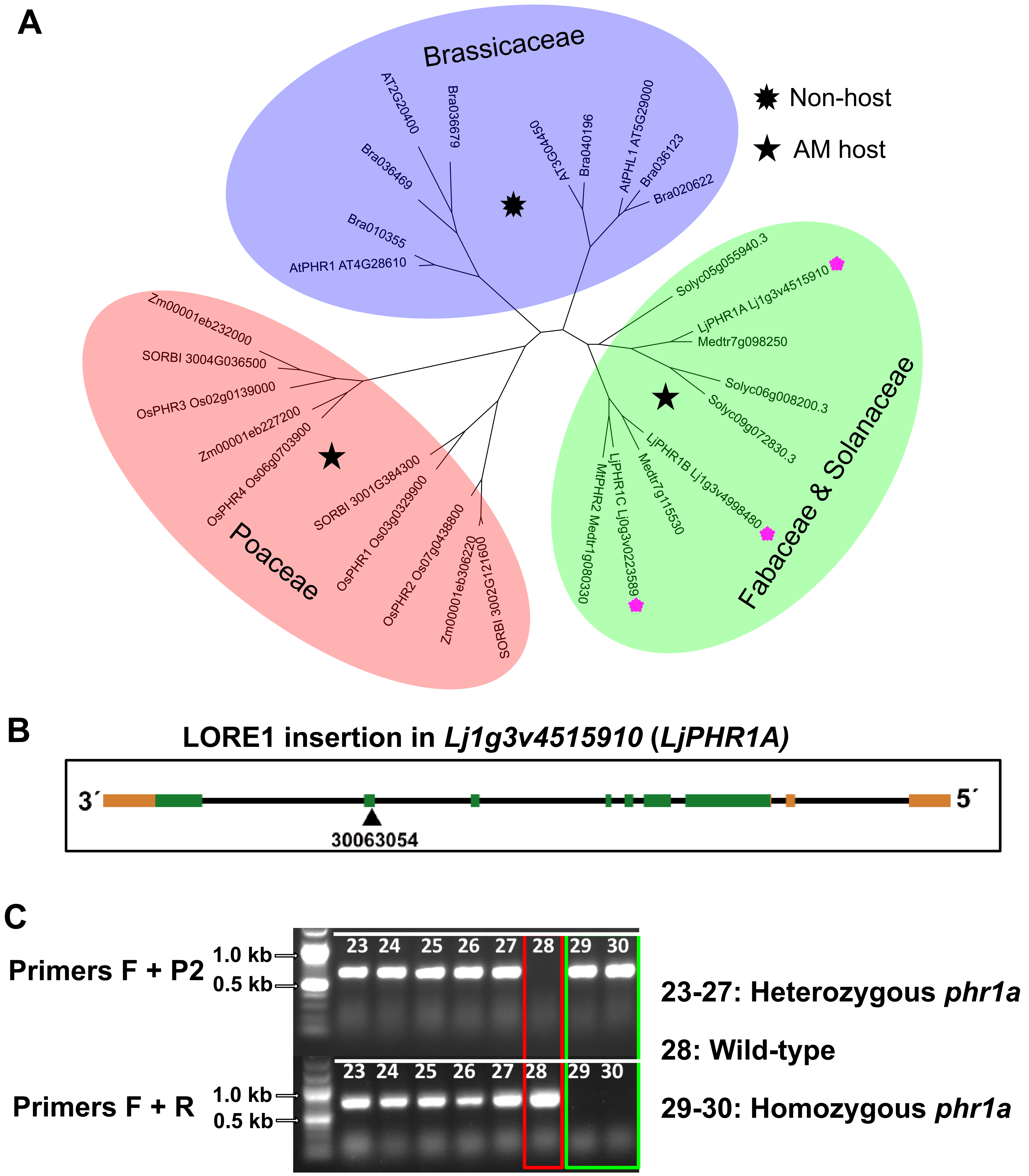

Supplementary Figure 19. Identification of *Lotus japonicus phr1a* mutant. (**A**) Phylogenetic tree of PHR proteins in representative *Brassicaceae, Poaceae, Fabaceae* and *Solanaceae*. The three *Lotus japonicus* PHR proteins are marked with pink stars. (**B**) Position of Lotus retrotransposon 1 (LORE1) insertion in *L. japonicus PHR1A*. The number indicates the Plant ID for LORE1 insertion. (**C**) Genotyping for LORE1 insertion in *phr1a*. The P2 primer sequence in located in the LORE1 insertion while F and R are *PHR1A* specific primers surrounding the insertion. 1 Kb PLus ladder of New England Biolabs (NEB, UK) was used as DNA ladder and 1 and 0.5 kb bands are shown as reference. The genotyping was performed once to select homozygous plants.

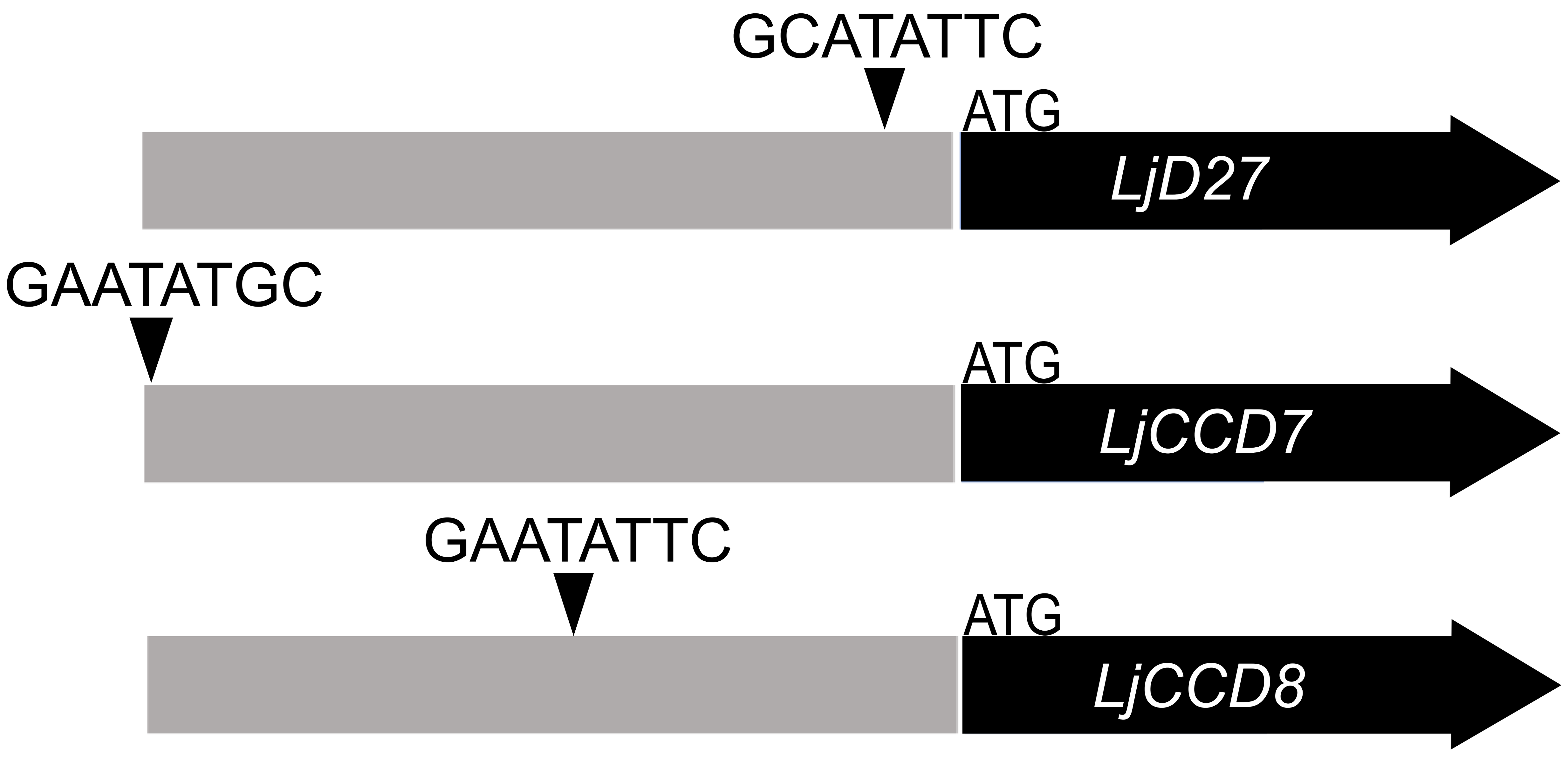

Supplementary Figure 20. Position of P1BS motifs in the promoters of strigolactone biosynthesis genes in *Lotus japonicus*. Promoter of length 1600 kb is represented in gray for each gene.

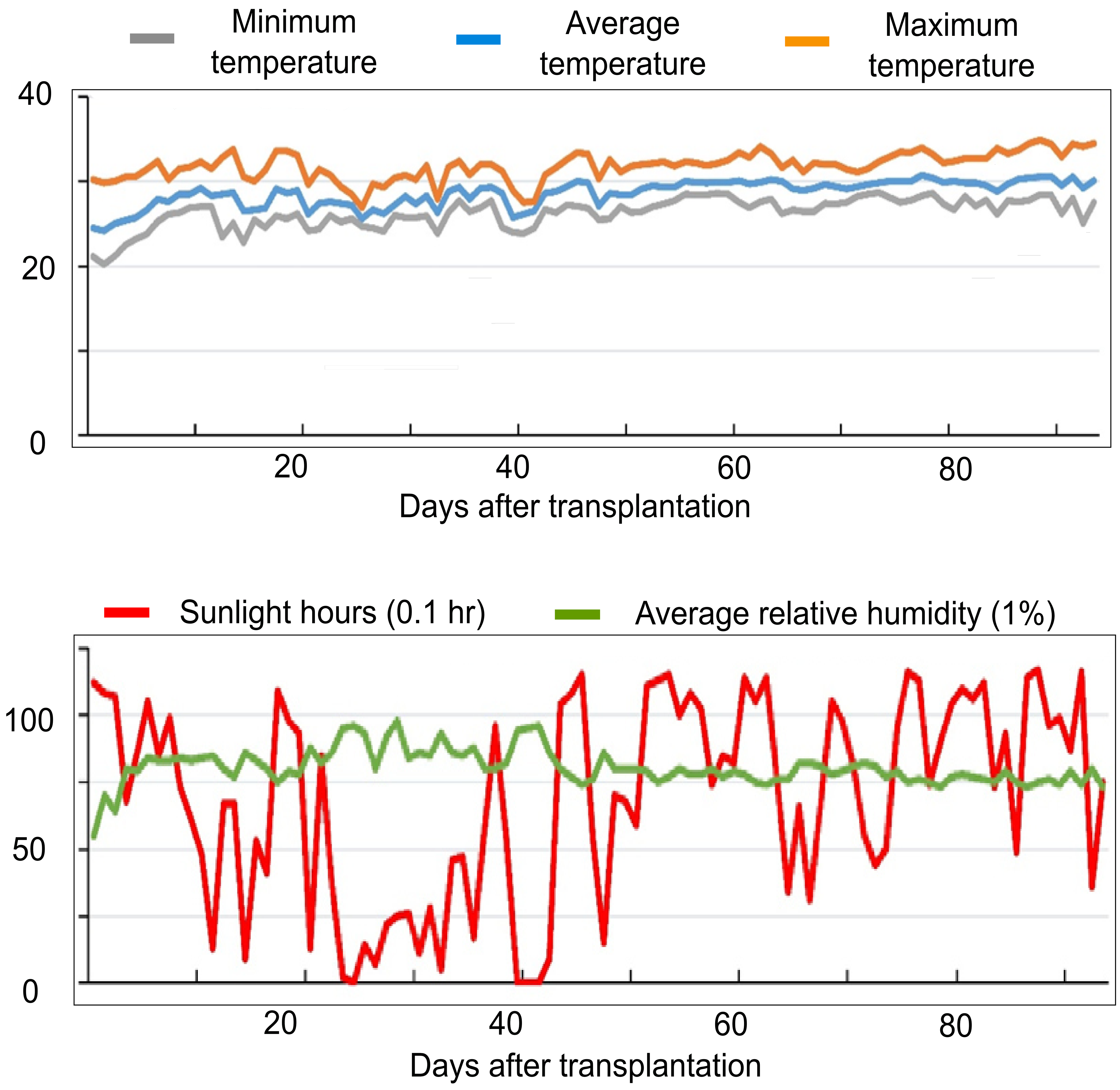

Supplementary Figure 21. Temperature, sunlight and relative humidity profiles during the greenhouse experiment in field soil.

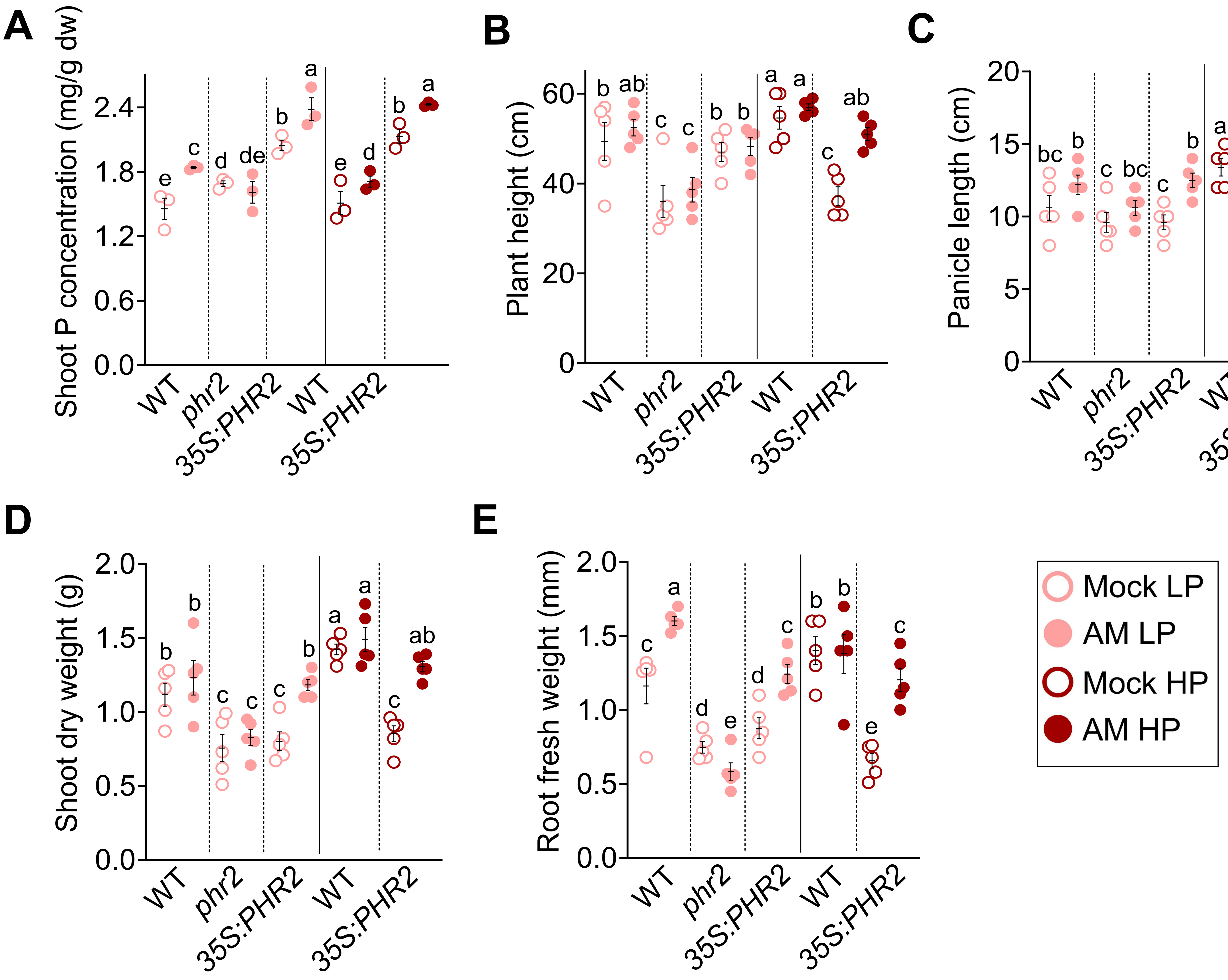

Supplementary Figure 22. PHR2 affects rice agronomic traits in a field soil. (**A**) Shoot phosphorus (P) concentration (mg/g dw), (**B**) Plant height (cm), (**C**) panicle length (cm), (**D**) shoot dry weight (g), (**E**) root fresh weight (g) in mock (Mock) and *R. irregularis* (AM) inoculated plants of wild type, *phr2* and *35S:PHR2* lines grown at LP (unfertilized) and of wild type and *35S:PHR2* lines grown at HP (fertilized with superphosphate fertilizer, P_2O_5). Traits were quantified for plants harvested at 110 days post transplanting into soil and inoculation. Statistics: Individual data-points and mean ± SE are shown. N=3-5 biologically independent samples; Brown-Forsythe and Welch's One-Way ANOVA test with Games-Howell's multiple comparison test was carried out. Different letters indicate statistical differences.

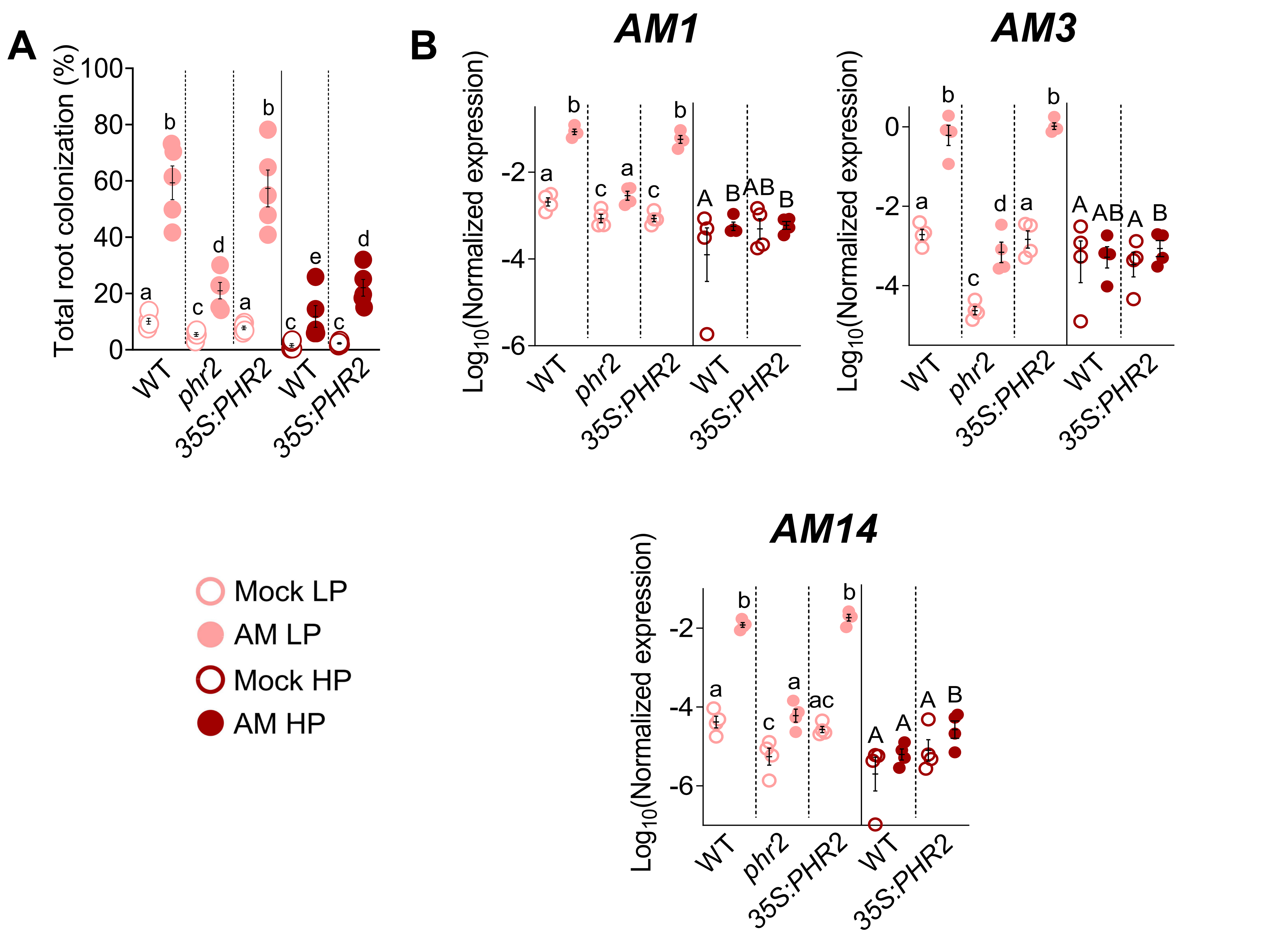

Supplementary Figure 23. Root colonization and RT-qPCR-based transcript accumulation of AM-marker genes in roots of plants grown in field soil. (**A**) Total root colonization (%) in *R. irregularis*-inoculated plants of wild type and *phr2* and *35S:PHR2* at LP (unfertilized) and in wild type and *35S:PHR2* lines at HP (fertilized with superphosphate fertilizer, P_2O_5). Roots were harvested at 110 days post transplantation into field soil and inoculation. Letters indicate statistical differences between genotypes, treatment and phosphate levels. Statistics: N=5; Kruskal-Wallis test with Dunn's posthoc comparison. (**B**) Relative transcript accumulation in mock inoculated (Mock) and *R.irregularis* colonized (AM) roots of the indicated genotypes grown in field soil and fertilized with LP or HP (as described in A). Expression values of indicated genes were normalized to the geometric mean of the expression of two housekeeping genes, *UBIQUITIN* and *CYCLOPHILIN*. Letters indicate statistical differences between genotypes and treatments within each phosphate level. Statistics: Individual data-points and mean ± SE are shown. (A) N=5 independent root systems; Brown-Forsythe and Welch's One-Way ANOVA test with Games-Howell's multiple comparisons test between genotypes and treatments. Different letters indicate statistical differences between genotypes and treatments. (B) N=3-4 biologically independent samples. Brown-Forsythe and Welch's One-Way ANOVA test with Games-Howell's multiple comparisons test was carried out for each phosphate level separately. Different letters indicate statistical differences between genotypes and treatments.

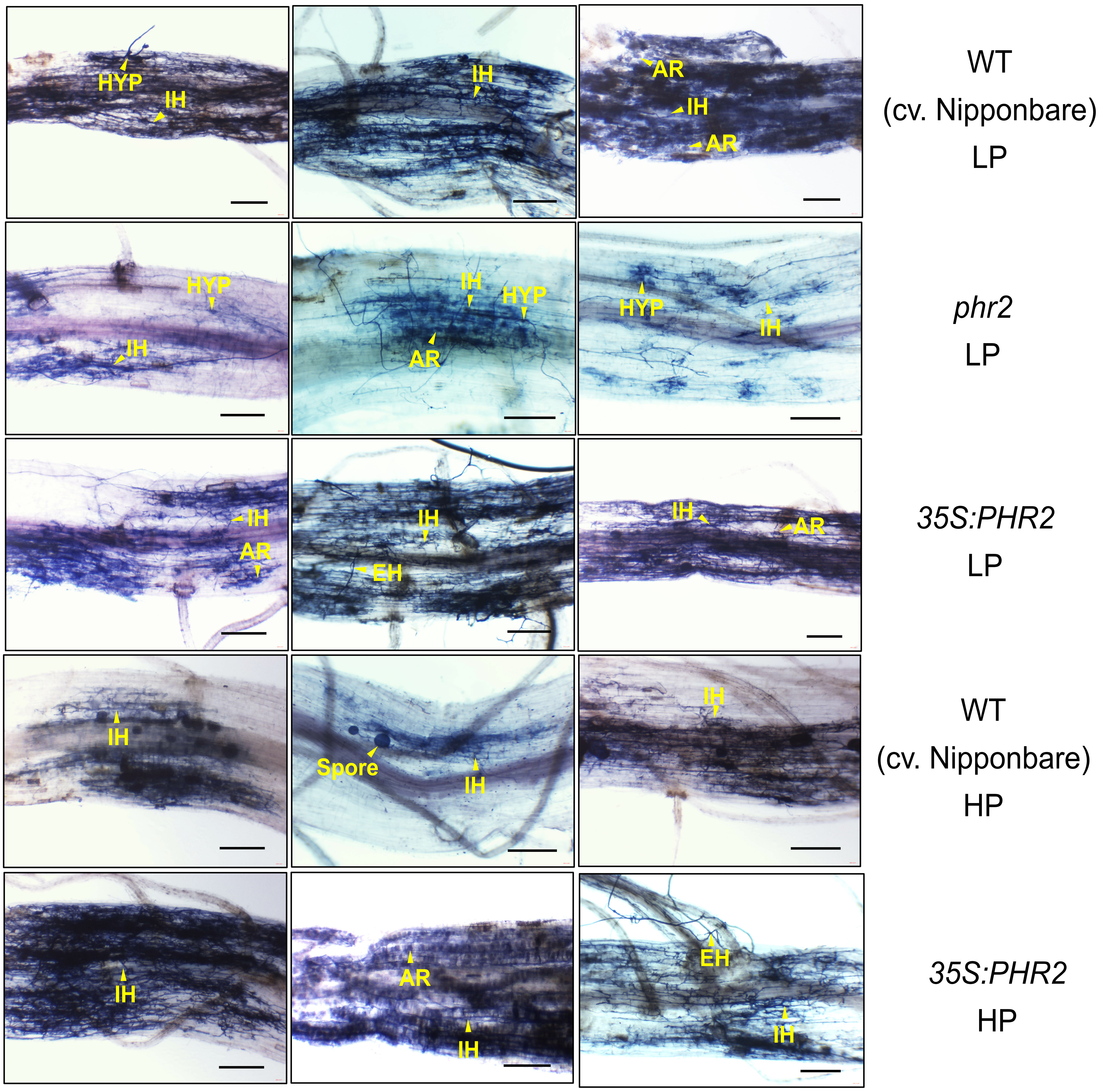

Supplementary Figure 24. Colonization of WT, *phr2* and *35S:PHR2*. Brightfield images of roots stained with acid-ink to visualize colonization of wild type (cv. Nipponbare), *phr2* and *35S:PHR2* roots by *R. irregularis* at 110 days post transplantation and grown at LP (unfertilized) or HP (fertilized with superphosphate fertilizer, P_2O_5) in field soil. Scale bars, 200 μm. Abbreviations: EH, extraradical hypha; HYP: hyphopodium; IH, intraradical hypha; AR, arbuscule, VE, vesicle. The phenotype was observed in root systems of 5 independent plants.

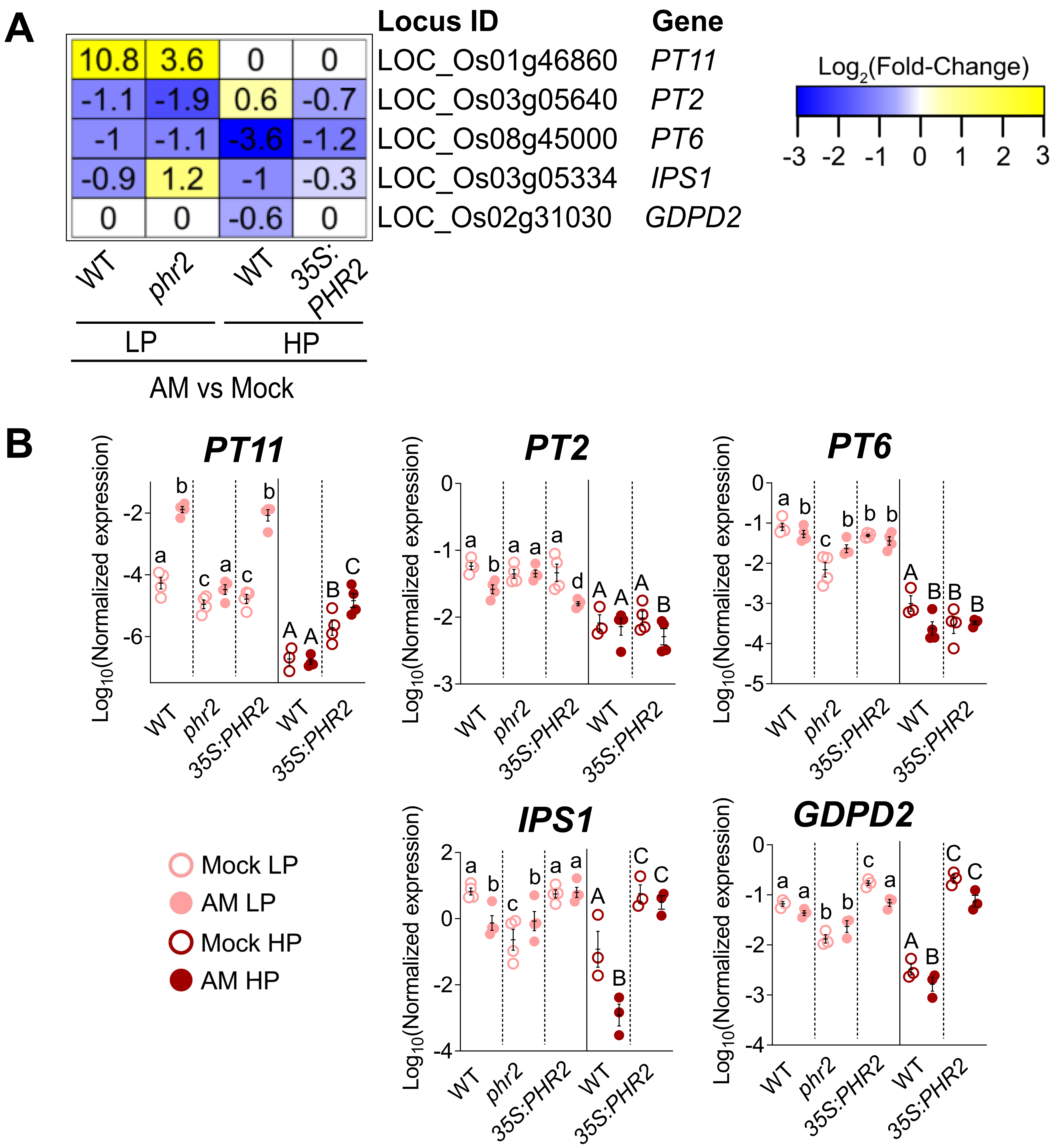

Supplementary Figure 25. RT-qPCR-based transcript accumulation of phosphate transporters and starvation marker genes in roots of plants grown in field soil. (**A**) RNA-seq-based fold-change of transcript accumulation (AM vs mock) of phosphate transporter genes involved in AM-mediated (*PT11*) or direct (*PT2*, *PT6*) P_i uptake, as well as the phosphate starvation marker genes (*IPS1*, *GDPD2*) at LP (25 µM P_i) or HP (500 µm P_i) in quartz sand. (**B**) RT-qPCR based relative transcript accumulation in mock inoculated (Mock) and *R. irregularis* colonized (AM) roots of the indicated genotypes grown in field soil and fertilized with LP or HP (as described in A) is shown. Roots were harvested at 110 days post transplanting and inoculation. Expression values of indicated genes were normalized to the geometric mean of the expression of two housekeeping genes, *UBIQUITIN* and *CYCLOPHILIN*. Letters indicate statistical differences between genotypes and treatments within each phosphate level. Statistics: Individual data-points and mean ± SE are shown. N=3-4 biologically independent samples. Brown-Forsythe and Welch's One-Way ANOVA test with Games-Howell's multiple comparisons test was carried out for each phosphate level separately. Different letters indicate statistical differences.

Supplementary Table 1. Primers used for RT-qPCR and genotyping.

Primer name	Forward Primer (5'→3')	Reverse Primer (5'→3')
CCD7-qRT	AATGCACTTGTGGCAAAACTAGAG	CATTGGAAAAGTGAGGTTCTTTGG
CCD8b-qRT	CAACTATGCCTTTTGGGTTAAAG	AAAGTCTCGGCCAAATCCT
DLK2C-qRT	CGATGTTGCCATATAGGTTGTGC	ACAAGGGAGCACACATGCAG
ZAS-qRT	TATGGAGGCCTTGCAAAACTTTGTC	CATTGTGTTTGCTAGTGATGATCTG
NSP2-qRT	TCAGCTGCTTCAACCACAGC	TGTTGGGACCCGTCTCCTC
CYCLOPS-qRT	GGTTTGGCTTGGTACAGCATCT	GGGAGGCAGGTCATCACAA
CCaMK-qRT	AGGCCAACAGCAAGTGATCT	CGCAGATTATCCAGCTCCTC
ABCB20-qRT	GAAATGCTTGATAGGGACACAC	TGAAACTCAGTTCTTCCCATGA
SYMRK-qRT	CCTGGCATAAAAGGGCAATA	GTGCTTTCGATGGACCTCAT
CERK1-qRT	TGGAATCGTGTACATCCCCG	CAAGTTGTGTGGAATCTTCAG
SLR1-qRT	GACGTCAACGAACGCTCAATT	CGGAGTCCAGTCGTCGATCT
PT11-qRT	ATATCCAAGGCCTCGTTCCT	CCGATCAGCTGGATCATGT
PT13-qRT	CAGGACGAGTATGGCCTCTT	TCGAGGACGAACCAACAGA
RLCK210 (KIN2)-qRT	CCTCATGGAGATGGACAAGAG	GATACCATCTCCTCCTCCAAAC
AM1-qRT	ACCGTGTGGGAGATGGAGTT	CCTGCAGCTCTTCCTCATCT
AM3-qRT	CTGTTGTTACATCTACGAATAAGGAGAAG	CAACTCTGGCCGGCAAGT
AM14-qRT	CCAACACCGTTGCAAGTACAATAC	GCACTTTGAAATTGGACTGTAAGAAA
PT2-qRT	GACGAGACCGCCCAAGAAG	TTTTCAGTCACTCACGTCGAGAC
PT6-qRT	CCGCCCCTGCAAACTGTA	CAACTGGCGGTTTCTTCGAT
OsUbiq-qRT	CATGGAGCTGCTGCTGTTCTAG	CAGACAACCATAGCTCCATTGG
CYCLOPHILIN2-qRT	AGCTCTCCTAGATCTGTGCTG	GCGATATCATAGAACGAGCGAC
IPS1-qRT	TTGGCAATTATTCGGTGGAT	ACCATTTCACCATCCTCTTTATG
GDPD2-qRT	GCCCAGTCATCTTCCATGATA	CCAATTCACTATCCGACCATCT
qPCR LjUbi	ATGCAGATCTTCGTCAAGACCTTG	ACCTCCCCTCAGACGAAG
qPCR LjPHR1A	CCGAATTGGAAGCATCCAAAGC	CTCGGAAGCTTGACTTTCGG
qPCR LjSYMRK	GAGGGTCAAAGGTGGATGA	GCGAACAATGGCGACCA
qPCR LjCCaMK	GGAGACAATGCAACTCTGTCTGA	CGGTGCTAGAGGGATCAATGA
qPCR LjCYCLOPS	GCTGGCAGATGAAAAAGAGC	GCGTGTTTGAGCACAACATT
qPCR LjD27	GCCATCTCAATCGTTTATCAAG	GCTTCAGTGCTGGATCATC
qPCR LjCCD7	GTATGGAGTGTTTAAGATGCCC	TAAAATGACTGCGTGGAAG
qPCR LjCCD8	GGACACGCTTAGGAAATTCG	TCTGTCACAATGGGATGTGC
LjPHR1A genotyping	TTGGTTATAAAGGACCGCAAG	TTCCTAACTAAGCTTGCCCATAA
LORE1 P2		CCATGGCGGTTCCGTGAATCTTAGG

Supplementary Table 2. Primers used for ChIP qPCR.
Primers with name appended with “motif”, “left” and “right” were used for amplifying sequences flanking P1BS (GNATATNC), or 1000 bp left and 1000 bp right of P1BS motif respectively (-1000 bp and +1000 bp in Fig. S17). In case of *ECA1*pro, primers with name appended with “motif” represents P1BS-like (AMATATYC). In case of *CERK1*pro, primers with names appended with “GAGAmotif” and “CTTC motif” were used for amplifying sequences amplifying GGAGAGGA and TCCTCTTGTTCTTC elements respectively, while primer with names appended with “GAGAleft” and “CTTCright” were used for amplifying sequences 1000 bp left of GAGA and 1000 bp right of CTTC respectively.

Primer name	Forward primer (5'→3')	Reverse primer (5'→3')
CERK1 pro-ChIP-motif	TCGCAGTTTACAGTCGGAATC	TTGGATATACGGGCACACATTTA
CERK1 pro-ChIP-left	GGCTGCTACATCACAAATTCAC	GGATGTGTTCGGCTGGTATT
CERK1 pro-ChIP-right	AAGAACACAGAGTGAGCTGTAA	GGGAAGAAAGGGAGAAGAAGAG
CCD7 pro-ChIP-motif	GGGCCTATAACTGCATATTCTCC	GTGCCCACGTAATTTGAAAGAG
CCD7 pro-ChIP-left	CCTTCACTTGGCGTTACAGA	CAGACACTAAACAGCACTACGA
CCD7 pro-ChIP-right	CATGCAGGTTCGTGGAGAC	TCACATTGCCCACCTTCTTC
ZAS pro-ChIP-motif	AGACACATGGATGCAGAGAAG	CGTGACGGATATTCCAAGATGA
ZAS pro-ChIP-left	CATTGGTGTTGCTGATGTTCTT	GCGGCCTACATTCTCAACTAT
ZAS pro-ChIP-right	GTCACATGGCATGCTACAAAC	AAATAACGGGTCCACCAATTTAAG
SYMRK pro-ChIP-motif	TGATTCCTCCCTTCCTCCTT	TTTCGTTCCGTGTCGTCATC
SYMRK pro-ChIP-left	ACAGTAACAAGGCTGAGTGTATC	AAGCAGCAATCCATCTACTCC
SYMRK pro-ChIP-right	TCTGCAGGACAACAACTTCA	GCAAATGGTAAAGAACAGCATCTA
ECA1 pro-ChIP-motif	CGCACAGCAGAGCACAA	CCATTCTCCACTCTCCGTTTC
ECA1 pro-ChIP-left	GAGATCGCATGGAACCGAAA	CTTCTCCTCTCTTCCGCATTG
ECA1 pro-ChIP-right	TCGTGTAGGACAGACCTTGAT	AATCCTAGTCACCAGTCCTACC
PT11 pro-ChIP-motif1	CGAGAGGAGAATGACGAAATCA	GCTCTTCTCCCATATCCATCAG
PT11 pro-ChIP-motif2	TGATTGGCGATTCCTACCATAC	GCGTAGCGGTAAATCGATGA
PT11 pro-ChIP-left	ATGCGCCACACGTAGTC	CCATGATCGTCTCTAGCATCTTC
PT11 pro-ChIP-right	AGCGGTGAAGCAGCAAA	CTCTAGATAAGTGGGACCGTACA
GDPD2 pro-ChIP-motif	TGCCTTTGGACCGGAATATC	AAGGAAGGAAGCGGGAATG
GDPD2 pro-ChIP-left	TGTGCTCTCGTGATGAATCTG	CTGTTCCACGACGGGTTAAA
GDPD2 pro-ChIP-right	TGAGCTGCTGTTCCGATTC	TGTCGATCGATTCGTATTTCCC
NSP2 pro-ChIP-motif	GCATTACGGGAAGCAACAAAG	GCTGAACTGCTGAAGACTGA
NSP2 pro-ChIP-left	CCATAGGTCGAGACTTGAGAG	CCCTTGGTACTTTAGAAATAGATGT
NSP2 pro-ChIP-right	GCCTTGGCAACAAAGCTAAG	AGAAATGTGCCGAGAGAGATG
CERK1 pro-ChIP-GAGAmotif	CAGTCCTGAACAGAGGACATAAG	GACTCCTCTCCAGACACTTCTA
CERK1 pro-ChIP-CTTCmotif	GGAGTCAAGGTTAGTGGCTAAG	CTGTGTTCTTTGCTTACGGATG
CERK1 pro-ChIP-GAGAleft	GTCTACCTCCACATGTCTCAAC	CCTAGGAAGAGGCCTAGATACA
CERK1 pro-ChIP-CTTCright	TACCCGGCCAACAACATC	TGAGGAACAGCCCGTAGT
RLCK210 pro-ChIP-motif	TTTGTGAATGAATTAGGTGCGT	GTGTTGTATGAGTATTGTCGAATGT
RLCK210 pro-ChIP-left	TACTATCACACCACGCGTCTA	GAGATTAGTAGATGGTCCCTGTAATTT
RLCK210 pro-ChIP-right	ACATGACCACCAGGCAAG	AATAAGACGGACGGTCAAACA

Supplementary Table 3. Primers used for cloning.

Purpose	Name	Sequence
c*PHR2* cloning for p*35S*:c*PHR2*	MP503	ATGAAGACTTTACGGGTCTCACACCATGGAGAGAATAAGCACCAATCAGC
	MP508	ATGAAGACTTCAGAGGTCTCACCTTTCTGTCACCTGATTCT GAAACAAAAATTTAAGG
p*GDPD2* cloning for p*GDPD2*:GUS	MP595	TTTGGTCTCAGCGGTGTTCATATATCTGATGTGACACGTC
	MP600	TTTGGTCTCACAGATATATTCGGAGGATGTCCTAGCTG
p*GDPD2m* cloning for p*GDPD2m*:GUS	MP595	TTTGGTCTCAGCGGTGTTCATATATCTGATGTGACACGTC
	MP596	TTGGTCTCACGCGGACGGTCCAAAGGCACGC
	MP597	TTGGTCTCACGCGAATGGAGGATAAACCATCCGATCCGC
	MP598	TTGGTCTCAGGTCCGCGGAGGGGTGGGGATGCGTTC
	MP599	TTGGTCTCAGACCCATTCCCGCTTCCTTC
	MP600	TTTGGTCTCACAGATATATTCGGAGGATGTCCTAGCTG
p*PT11* cloning for p*PT11*:GUS	MP609	TTGGTCTCTGCGGGGAGCAATAGACGAGGGATGCC
	MP614	TTGGTCTCTCAGACTCCGATGATGCCGTCGATCG
p*PT11m* cloning for p*PT11m*:GUS	MP609	TTGGTCTCTGCGGGGAGCAATAGACGAGGGATGCC
	MP610	TTGGTCTCTTACGCGGAGGTAAATACATGAAAAATTAA AAGTTAGTTAGC
	MP611	TTGGTCTCTCGTACACTGAACTACCCATTCACACC
	MP612	TTGGTCTCTATTCGCGGAGGCAGATAATCATGATTG
	MP613	TTGGTCTCTGAATACCAAAAACGACGCATTTCCGTCC
	MP614	TTGGTCTCTCAGACTCCGATGATGCCGTCGATCG
p*CCD7* cloning for p*CCD7*:GUS	MP549	TTTGGTCTCAGCGGGGGCGTGCACACTGCAAGCATC
	MP550	TTTGGTCTCAATGATGTCTGCAAGGACCCAGAGCTCTAC
	MP551	TTTGGTCTCATCATTCCTCTGTTCTTTCCACC
	MP552	TTTGGTCTCACAGACTTTGGACTTGGCCTCCTTC
p*CCD7m* cloning for p*CCD7m*:GUS	MP549	TTTGGTCTCAGCGGGGGCGTGCACACTGCAAGCATC
	MP591	TTTGGTCTCATCCGCGTAAGTTATAGGCCCCGTTCGTTTTGG ATTTTGATGGCACATTTTTC
	MP592	TTTGGTCTCACGGATCCGGGGAAAAATATTGAACTGGAATTAG
	MP552	TTTGGTCTCACAGACTTTGGACTTGGCCTCCTTC
p*ZAS* cloning for p*ZAS*:GUS	MP545	TTTGGTCTCAGCGGATATTTGGATGGTATGCAAAGCACATG
	MP546	TTTGGTCTCAAGTTACGTACTCCCTCTGTTTCAC
	MP547	TTTGGTCTCAAACTACGTACATATACCTAACGTAAC
	MP548	TTTGGTCTCACAGATCTGCTAGTAAAAAAAGCCTAAATCC
p*ZASm* cloning for p*ZASm*:GUS	MP545	TTTGGTCTCAGCGGATATTTGGATGGTATGCAAAGCACATG
	MP585	TTTGGTCTCAGTACTCAGAAAAAAATTTCCGTCCCTTGTCC
	MP586	TTTGGTCTCAGTACGCGGACAACGGGTCGTAGTCTTTAGTTATC
	MP587	TTTGGTCTCATACGCGCAATAAAAAAGACGACAAAAAAAT ACATCATAAAAATCGATG
	MP588	TTTGGTCTCACGTAGCATGGTTTTTTTCTTTTCTTTCCAG
	MP589	TTTGGTCTCAGAAGTCACGGAACCATCTTGGTG
	MP590	TTTGGTCTCACTTCGCGGACAAGATGACAAATGGAATTTCATCAC
	MP548	TTTGGTCTCACAGATCTGCTAGTAAAAAAAGCCTAAATCC

Supplementary Table 4. Plasmids used in this study. Produced by Golden Gate cloning (Level I, II and III). EV, empty vector; HR, hairy root; trafo, transformation.

**Purpose**	**Name**	**Description**
Golden Gate level I (LI) elements		
pMP900	LI *cPHR2*	PCR amplification of *OsPHR2* coding sequence from Nipponbare cDNA with MP503 + MP508 and assembly by BpiI cut ligation into LI pUC57 plasmid (BB03).
pPP3	LI C-D *GUS*	Supplementary reference 34
Golden Gate level II (LII) plasmids		
pMP903	LII F 3-4 p*35S:cPHR2*	Assembled by BsaI cut ligation from:<br>LI A-C p*35S* (G009) + LI dy B-C (BB6) + LI c*PHR2* + LI D-E c-Myc (G070) + LI E-F 35S-T (G059) + LI dy F-G (BB09) + LII R 3-4 (BB24)
pPP101	LIIc F 1-2 p*Ubi:mCherry*	Assembled by BsaI cut ligation from:<br>LI A-B p*Ubi* (G007) + LI B-C (BB06) dy + LI C-D *mCherry* (G023) + LI D-E (BB08) dy + LI E-F 35S-T (G059) + LI F-G dy (BB09) + LIIc F 1-2 (BB30) (Supplementary reference 34)
pPP28	LIIc R 5-6 p*35S :mCherry*	Assembled by BsaI cut ligation from:<br>LI A-B p*35S* (G009) + LI B-C (BB06) dy + LI C-D *mCherry* (G023) + LI D-E (BB08) dy + LI E-F 35S-T (G059) + LI F-G dy (BB09) + LIIc R 5-6 (BB30)<br>(Pimprikar et al. 2016)
pPP22	LIIc F 3-4 pOI:*GUS*	Assembled by BsaI cut ligation from:<br>LI A-B Esp3I-*lacZ* dy (G082) + LI B-C dy (BB06) + LI C-D *GUS* + LI D-E dy (BB08) + LI nos-T (G006) + LI F-G dy (BB09) + LIIc F 3-4 (BB33)
pMP200	LII F 5-6 p*Ubi:mCherry*	Assembled by BsaI cut ligation from:<br>LI A-B p*Ubi* (G007) + LI B-C (BB06) dy + LI C-D *mCherry* (G023) + LI D-E (BB08) dy + LI E-F 35S-T (G059) + LI F-G dy (BB09) + LII F 5-6 (BB28)
Golden Gate level III (LIII) plasmids for plant transformation		
pMP906<br>Overexpression of *OsPHR2 in N. benthamiana* leaves	LIIIβ F A-B p*35S:cPHR2*	Assembled by BpiI cut ligation from:<br>LII dy 1-2 (BB63) + LII dy 2-3 (BB39) + LII F 3-4 p*35S:cPHR2* + LII dy 4-5 ins (BB44) + LIIc R 5-6 p*35S :mCherry* + LIIIβ F A-B (BB53)
pMP301<br>Esp3I compatible destination backbone for localization of promoter activity	Esp3I cut ligation compatible backbone: LIIIβ fin<br>p*Ubi:mCherry_* pOI:*GUS* Esp3I	Assembled by BpiI cut ligation from:<br>LIIc F 1-2 p*Ubi:mCherry* + LII 2-3 ins (BB43) + LIIc F 3-4 pOI:*GUS* + LII dy 4-6 (BB41) + LIIIβ fin (BB52)
pMP302<br>BsaI compatible destination backbone for Localization of promoter activity	BsaI cut ligation compatible backbone: LIIIβ fin<br>p*Ubi:mCherry_* pOI:*GUS* BsaI	Assembled by Esp3I cut ligation from:<br>LIIIβ fin p*Ubi:mCherry_* pOI:*GUS* Esp3I + LI A-B Esp3I-ccdB dy (G084)

pMP909		Assembled by BsaI cut ligation from:
Transactivation of p*GDPD* :GUS in *N. benthamiana* leaves	LIIIβ fin	LIIIβ fin p*Ubi:mCherry_* pOI:*GUS* BsaI +
	p*Ubi* :*mCherry* _p*GDPD* :*GUS*	
		PCR amplicon MP595 + MP600 amplified from Nipponbare genomic DNA
pMP910		Assembled by BsaI cut ligation from:
Transactivation of p*GDPDm* :GUS in *N. benthamiana* leaves	LIIIβ fin	LIIIβ fin p*Ubi:mCherry_* pOI:*GUS* BsaI +
	p*Ubi* :*mCherry* _p*GDPDm* :*GUS*	PCR amplicons MP595 + MP596, MP597 + MP598, MP599 + MP600 amplified from LIIIβ fin p*Ubi* :*mCherry* _p*GDPD* :*GUS*
pMP911		Assembled by BsaI cut ligation from:
Transactivation of p*PT11* :GUS in *N. benthamiana* leaves	LIIIβ fin	LIIIβ fin p*Ubi:mCherry_* pOI:*GUS* BsaI +
	p*Ubi* :*mCherry* _p*PT11* :*GUS*	PCR amplicon MP609 + MP614 amplified from Nipponbare genomic DNA
pMP912		Assembled by BsaI cut ligation from:
Transactivation of p*PT11m* :GUS in *N. benthamiana* leaves	LIIIβ fin	LIIIβ fin p*Ubi:mCherry_* pOI:*GUS* BsaI +
	p*Ubi* :*mCherry* _p*PT11m* :*GUS*	PCR amplicons MP609 + MP610, MP611 + MP612, MP613 + MP614 amplified from LIIIβ fin p*Ubi* :*mCherry* _p*PT11* :*GUS*
pMP913		Assembled by BsaI cut ligation from:
Transactivation of p*CCD7* :GUS in *N. benthamiana* leaves	LIIIβ fin	LIIIβ fin p*Ubi:mCherry_* pOI:*GUS* BsaI +
	p*Ubi* :*mCherry* _p*CCD7* :*GUS*	PCR amplicons MP549 + MP550, MP551 + MP552 amplified from Nipponbare genomic DNA
pMP914		Assembled by BsaI cut ligation from:
Transactivation of p*CCD7m* :GUS in *N. benthamiana* leaves	LIIIβ fin	LIIIβ fin p*Ubi:mCherry_* pOI:*GUS* BsaI +
	p*Ubi* :*mCherry* _p*CCD7m* :*GUS*	PCR amplicons MP549 + MP591, MP592 + MP552 amplified from LIIIβ fin p*Ubi* :*mCherry* _p*CCD7* :*GUS*
pMP915		Assembled by BsaI cut ligation from:
Transactivation of p*ZAS* :GUS in *N. benthamiana* leaves	LIIIβ fin	LIIIβ fin p*Ubi:mCherry_* pOI:*GUS* BsaI +
	p*Ubi* :*mCherry* _p*ZAS* :*GUS*	PCR amplicons MP545 + MP546, MP547 + MP548 amplified from Nipponbare genomic DNA
pMP916		Assembled by BsaI cut ligation from:
Transactivation of p*ZASm* :GUS in *N. benthamiana* leaves	LIIIβ fin	LIIIβ fin p*Ubi:mCherry_* pOI:*GUS* BsaI +
	p*Ubi* :*mCherry* _p*ZASm* :*GUS*	PCR amplicons MP545 + MP585, MP586 + MP587, MP588 + MP589, MP590 + MP548 amplified from LIIIβ fin p*Ubi* :*mCherry* _p*ZAS* :*GUS*

Supplementary Table 5. Table for composition of buffer used in the transactivation assay.

Buffer composition for transactivation assay		
components	extraction buffer	assay buffer
$NaPO_4$	50 mM	50 mM
ß-mercaptoethanol	10 mM	10 mM
Na_2-EDTA	10 mM	10 mM
Triton X-100	0,1%	0,1%
N-laurylsarcosine	0,1%	0,1%
cOmplete(TM), EDTA-free Protease Inhibitor, Sigma-Aldrich (USA)	1x	-
Methylumbelliferyl-β-D-glucuronic acid dihydrate (MUG), Biosynth (USA)	-	1 mM

Supplementary References

1. Lota, F. *et al.* The cis-acting CTTC-P1BS module is indicative for gene function of *LjVTI12*, a Qb-SNARE protein gene that is required for arbuscule formation in *Lotus japonicus*. *The Plant Journal* **74**, 280-293, doi:10.1111/tpj.12120 (2013).
2. Roth, R. *et al.* A rice Serine/Threonine receptor-like kinase regulates arbuscular mycorrhizal symbiosis at the peri-arbuscular membrane. *Nature Communications* **9**, 1-12 (2018).
3. Ao, Y. *et al.* OsCERK1 and OsRLCK176 play important roles in peptidoglycan and chitin signaling in rice innate immunity. *The Plant Journal* **80**, 1072-1084 (2014).
4. Buendia, L., Girardin, A., Wang, T., Cottret, L. & Lefebvre, B. LysM receptor-like kinase and LysM receptor-like protein families: an update on phylogeny and functional characterization. *Frontiers in Plant Science* **9**, 1531 (2018).
5. Capoen, W. *et al.* Nuclear membranes control symbiotic calcium signaling of legumes. *Proceedings of the National Academy of Sciences U.S.A* **108**, 14348-14353 (2011).
6. Chiu, C. H., Choi, J. & Paszkowski, U. Independent signalling cues underpin arbuscular mycorrhizal symbiosis and large lateral root induction in rice. *New Phytologist* **217**, 552-557 (2018).
7. Chen, C., Gao, M., Liu, J. & Zhu, H. Fungal symbiosis in rice requires an ortholog of a legume common symbiosis gene encoding a Ca^{2+}/calmodulin-dependent protein kinase. *Plant Physiology* **145**, 1619-1628 (2007).
8. Takeda, N., Tsuzuki, S., Suzaki, T., Parniske, M. & Kawaguchi, M. *CERBERUS* and *NSP1* of *Lotus japonicus* are common symbiosis genes that modulate arbuscular mycorrhiza development. *Plant and Cell Physiology* **54**, 1711-1723 (2013).
9. Delaux, P. M., Bécard, G. & Combier, J. P. NSP1 is a component of the Myc signaling pathway. *New Phytologist* **199**, 59-65 (2013).
10. Liu, W. *et al.* Strigolactone biosynthesis in *Medicago truncatula* and rice requires the symbiotic GRAS-type transcription factors NSP1 and NSP2. *The Plant Cell* **23**, 3853-3865 (2011).
11. Yano, K. *et al.* Function and evolution of a *Lotus japonicus* AP2/ERF family transcription factor that is required for development of infection threads. *DNA Research* **24**, 193-203 (2017).
12. Saito, K. *et al.* NUCLEOPORIN85 is required for calcium spiking, fungal and bacterial symbioses, and seed production in *Lotus japonicus*. *The Plant Cell* **19**, 610-624 (2007).
13. Zhang, X., Pumplin, N., Ivanov, S. & Harrison, M. J. EXO70I is required for development of a sub-domain of the periarbuscular membrane during arbuscular mycorrhizal symbiosis. *Current Biology* **25**, 2189-2195 (2015).
14. Wang, E. *et al.* A common signaling process that promotes mycorrhizal and oomycete colonization of plants. *Current Biology* **22**, 2242-2246 (2012).
15. Keymer, A. *et al.* Lipid transfer from plants to arbuscular mycorrhiza fungi. *Elife* **6**, e29107 (2017).
16. Wang, S. *et al.* Functional analysis of the OsNPF4.5 nitrate transporter reveals a conserved mycorrhizal pathway of nitrogen acquisition in plants. *Proceedings of the National Academy of Sciences U.S.A* **117**, 16649-16659 (2020).
17. Xue, L. *et al.* Network of GRAS transcription factors involved in the control of arbuscule development in *Lotus japonicus*. *Plant Physiology* **167**, 854-871 (2015).

18. Park, H.-J., Floss, D. S., Levesque-Tremblay, V., Bravo, A. & Harrison, M. J. Hyphal branching during arbuscule development requires *Reduced Arbuscular Mycorrhiza1*. *Plant Physiology* **169**, 2774-2788 (2015).
19. Yano, K. *et al.* CYCLOPS, a mediator of symbiotic intracellular accommodation. *Proceedings of the National Academy of Sciences U.S.A* **105**, 20540-20545 (2008).
20. Chen, C., Ané, J. M. & Zhu, H. OsIPD3, an ortholog of the Medicago truncatula DMI3 interacting protein IPD3, is required for mycorrhizal symbiosis in rice. *New Phytologist* **180**, 311-315 (2008).
21. Pumplin, N. *et al.* Medicago truncatula Vapyrin is a novel protein required for arbuscular mycorrhizal symbiosis. *The Plant Journal* **61**, 482-494 (2010).
22. Feddermann, N. *et al.* The *PAM1* gene of petunia, required for intracellular accommodation and morphogenesis of arbuscular mycorrhizal fungi, encodes a homologue of *VAPYRIN*. *The Plant Journal* **64**, 470-481 (2010).
23. Chen, C., Fan, C., Gao, M. & Zhu, H. Antiquity and function of CASTOR and POLLUX, the twin ion channel-encoding genes key to the evolution of root symbioses in plants. *Plant Physiology* **149**, 306-317 (2009).
24. Floss, D. S., Schliemann, W., Schmidt, J., Strack, D. & Walter, M. H. RNA interference-mediated repression of *MtCCD1* in mycorrhizal roots of *Medicago truncatula* causes accumulation of C27 apocarotenoids, shedding light on the functional role of CCD1. *Plant Physiology* **148**, 1267-1282 (2008).
25. Floß, D. S. *et al.* Knock-down of the MEP pathway isogene *1-deoxy-D-xylulose 5-phosphate synthase 2* inhibits formation of arbuscular mycorrhiza-induced apocarotenoids, and abolishes normal expression of mycorrhiza-specific plant marker genes. *The Plant Journal* **56**, 86-100 (2008).
26. Doidy, J. *et al.* The *Medicago truncatula* sucrose transporter family: characterization and implication of key members in carbon partitioning towards arbuscular mycorrhizal fungi. *Molecular Plant* **5**, 1346-1358 (2012).
27. Wang, C. *et al.* OsCERK1-mediated chitin perception and immune signaling requires receptor-like cytoplasmic kinase 185 to activate an MAPK cascade in rice. *Molecular Plant* **10**, 619-633 (2017).
28. Xiu-mei, W. *et al.* Identification and cloning of tillering-related genes *OsMAX1* in rice. *Rice Science* **22**, 255-263 (2015).
29. Ho-Plágaro, T. *et al.* DLK2 regulates arbuscule hyphal branching during arbuscular mycorrhizal symbiosis. *New Phytologist* **229**, 548-562 (2021).
30. Krajinski, F. *et al.* The H^+-ATPase HA1 of *Medicago truncatula* is essential for phosphate transport and plant growth during arbuscular mycorrhizal symbiosis. *The Plant Cell* **26**, 1808-1817 (2014).
31. Wang, E. *et al.* A H^+-ATPase that energizes nutrient uptake during mycorrhizal symbioses in rice and *Medicago truncatula*. *The Plant Cell* **26**, 1818-1830 (2014).
32. Yu, N. *et al.* A DELLA protein complex controls the arbuscular mycorrhizal symbiosis in plants. *Cell Research* **24**, 130-133 (2014).
33. Nadal, M. *et al.* An N-acetylglucosamine transporter required for arbuscular mycorrhizal symbioses in rice and maize. *Nature Plants* **3**, 1-7 (2017).
34. Pimprikar, P. *et al.* A CCaMK-CYCLOPS-DELLA complex activates transcription of *RAM1* to regulate arbuscule branching. *Current Biology* **26**, 987-998 (2016).
