# Supplementary material for: PHOSPHATE STARVATION RESPONSE transcription factors enable arbuscular mycorrhiza symbiosis
Source: Nat Commun. 2022 Jan 25;13:477. doi: 10.1038/s41467-022-27976-8 (PMC8789775; doi:10.1038/s41467-022-27976-8)
Supplement: Supplementary file 3 — Description of Additional Supplementary Files [file 41467_2022_27976_MOESM3_ESM.pdf]

Description of Additional Supplementary Files

File name: Supplementary Data 1

Description: RNA-seq based expression Data for all genes expressed in Mock (not inoculated) or AM (*Rhizophagus irregularis*-inoculated) roots of WT, *phr2* and *35S*:*PHR2*.

File name: Supplementary Data 2

Description: Information on clustering analysis for the genes significantly regulated in the Mock roots of *phr2* vs WT grown at LP and *35S*:*PHR2* vs WT grown at HP samples in RNASeq.

File name: Supplementary Data 3

Description: List of DEGs upregulated in wild type AM vs Mock LP and DEGs downregulated in *phr2* vs WT LP Mock roots.

File name: Supplementary Data 4 Description: List of AM genes (AM genelist). File name: Supplementary Data 5

Description: Clustering analysis for the combined unique list of DEGs (14333 DEGs) significantly regulated in AM vs Mock samples of *phr2* under low phosphate (LP), *35S*:*PHR2* under high phosphate (HP) and WT under both phosphate conditions.

File name: Supplementary Data 6

Description: Genes with reduced expression in *phr2* vs WT in AM and/or Mock samples at LP and required for or involved in AM symbiosis as determined by mutant analysis (red bold), RNAi (green) or overexpression (blue).

File name: Supplementary Data 7

Description: Venn intersection of DEGs downregulated in *phr2* vs WT in AM and/or Mock samples at LP with DEGs upregulated in *smax1* or *d3*/*smax1* vs WT (Choi et al., 2020) and with AM genelist (Supplementary Data 4).

File name: Supplementary Data 8

Description: List of genes annotated to PHR2 binding sites (detected as MACS2 narrow peaks) and fasta sequence for PHR2 binding sites in ChIP-Seq of PHR2-FLAG.

File name: Supplementary Data 9

Description: Occurrence analysis for *P1BS*/*P1BS*-like motifs in 3000 bp upstream sequence for genes with reduced expression in *phr2* and PHR2 targets in the two ChIP biological replicates.

File name: Supplementary Data 10

Description: PHR2 targets as identified by ChIP-Seq and RNASeq assays used in this work, therefore Set A + B genes in Fig. 3A and ChIP binding statistics for known targets of PHR2.
