# Supplementary material for: PHOSPHATE STARVATION RESPONSE transcription factors enable arbuscular mycorrhiza symbiosis
Source: Nat Commun. 2022 Jan 25;13:477. doi: 10.1038/s41467-022-27976-8 (PMC8789775; doi:10.1038/s41467-022-27976-8)
Supplement: Supplementary file 14 — Reporting Summary [file 41467_2022_27976_MOESM14_ESM.pdf]

nature portfolio

Corresponding author(s): Caroline Gutjahr, Moxian Chen, Jianhua Zhang

Last updated by author(s): Dec 6, 2021

Reporting Summary

Nature Portfolio wishes to improve the reproducibility of the work that we publish. This form provides structure for consistency and transparency in reporting. For further information on Nature Portfolio policies, see our Editorial Policies and the Editorial Policy Checklist.

Statistics

For all statistical analyses, confirm that the following items are present in the figure legend, table legend, main text, or Methods section.

n/a	Confirmed	
☐	☒	The exact sample size (*n*) for each experimental group/condition, given as a discrete number and unit of measurement
☐	☒	A statement on whether measurements were taken from distinct samples or whether the same sample was measured repeatedly
☐	☒	The statistical test(s) used AND whether they are one- or two-sided<br>*Only common tests should be described solely by name; describe more complex techniques in the Methods section.*
☒	☐	A description of all covariates tested
☐	☒	A description of any assumptions or corrections, such as tests of normality and adjustment for multiple comparisons
☐	☒	A full description of the statistical parameters including central tendency (e.g. means) or other basic estimates (e.g. regression coefficient) AND variation (e.g. standard deviation) or associated estimates of uncertainty (e.g. confidence intervals)
☐	☒	For null hypothesis testing, the test statistic (e.g. *F*, *t*, *r*) with confidence intervals, effect sizes, degrees of freedom and *P* value noted<br>*Give P values as exact values whenever suitable.*
☒	☐	For Bayesian analysis, information on the choice of priors and Markov chain Monte Carlo settings
☒	☐	For hierarchical and complex designs, identification of the appropriate level for tests and full reporting of outcomes
☒	☐	Estimates of effect sizes (e.g. Cohen's *d*, Pearson's *r*), indicating how they were calculated

Our web collection on statistics for biologists contains articles on many of the points above.

Software and code

Policy information about availability of computer code

Data collection	Zeiss ZEN 2020 v3.3 & Swift Easy View (AM imaging); CFX Maestro Software 2.0 (RT-qPCR)
Data analysis	SALMON 0.7.0, and Bioconductor 3.13 (RNASeq); TRIM-Galore 0.6.4, BWA-MEM2, samtools-1.14, bedtools 2.29.2, deepTools 2.0, MACS2, IGV 2.8.9 (ChIP-Seq); GraphPad Prism v8.0 (AM colonization, qPCR, transactivation assay, field soil assay), Clustal Omega 1.2.4 (Phylogenetic analysis)

For manuscripts utilizing custom algorithms or software that are central to the research but not yet described in published literature, software must be made available to editors and reviewers. We strongly encourage code deposition in a community repository (e.g. GitHub). See the Nature Portfolio guidelines for submitting code & software for further information.

Data

Policy information about availability of data

All manuscripts must include a data availability statement. This statement should provide the following information, where applicable:

- Accession codes, unique identifiers, or web links for publicly available datasets
- A description of any restrictions on data availability
- For clinical datasets or third party data, please ensure that the statement adheres to our policy

The RNAseq data generated in this study have been deposited in the SRA database under BioProject accession code PRJNA735781:
https://Dataview.ncbi.nlm.nih.gov/object/PRJNA735781?reviewer=3cvg1gepckl1es04rfuslpiad2
The ChIP-Seq data generated in this study have been deposited in the SRA database under BioProject accession code PRJNA735744:

https://Dataview.ncbi.nlm.nih.gov/object/PRJNA735744?reviewer=hjvlce636jnenf75q90rcce5a0
Source data are provided with this paper.
Rice reference transcriptome was downloaded from publicly accessible Phytozome v12 database (https://phytozome.jgi.doe.gov/pz/portal.html).

Field-specific reporting

Please select the one below that is the best fit for your research. If you are not sure, read the appropriate sections before making your selection.

☒ Life sciences ☐ Behavioural & social sciences ☐ Ecological, evolutionary & environmental sciences

For a reference copy of the document with all sections, see nature.com/documents/nr-reporting-summary-flat.pdf

Life sciences study design

All studies must disclose on these points even when the disclosure is negative.

Sample size	No statistical analyses were performed to predetermine sample size in any of the experiment. Sample sizes were chosen according to the standard for these types of analyses. In most experiments, 3-5 biological replicates (represented by one plant each) were used. In transactivation assays, 11-12 biological replicates were collected. These represent 11-12 independent Agrobacterium infiltrations, one per leaf into leaves of 4 individual plants. In ChIP-Seq, 2 independent replicate experiments were conducted, which is currently the minimum standard sample size for ChIP-Seq assays.
Data exclusions	No data presented or mentioned in this work has been excluded.
Replication	For lab-based AM phenotyping and field soil assay, 3-5 biological replicates each from different plants from same experiment were used. For qPCR, 3 biological replicates from same experiment were used. For RNASeq, 3 biological replicates from same experiment were used. For transactivation assays, 11-12 biological replicates from 4 individual plants from same experiment were used. For ChIP-Seq, 2 biological replicates collected from 2 different replicate experiments were used. The experiment in field soil confirms the experiments performed in the laboratory and therefore act as an independent confirmation.
Randomization	Samples were randomly allocated to each group and treated.
Blinding	For AM colonization quantification, investigators were blinded. For qPCR, RNASeq and ChIP-Seq, an unbiased quantification was made and hence investigators were not blinded. For qPCR, RNASeq and ChIP-seq, templates were prepared by the first-author and an organized pipetting scheme was necessary not to mix up different samples. Furthermore, transcript or DNA quantity in qPCR, RNASeq and ChIP-seq was measured by a machine, so there was no influence of the experimenter.

Reporting for specific materials, systems and methods

We require information from authors about some types of materials, experimental systems and methods used in many studies. Here, indicate whether each material, system or method listed is relevant to your study. If you are not sure if a list item applies to your research, read the appropriate section before selecting a response.

Materials & experimental systems

n/a	Involved in the study	
☐	☒	Antibodies
☒	☐	Eukaryotic cell lines
☒	☐	Palaeontology and archaeology
☒	☐	Animals and other organisms
☒	☐	Human research participants
☒	☐	Clinical data
☒	☐	Dual use research of concern

Methods

n/a	Involved in the study	
☐	☒	ChIP-seq
☒	☐	Flow cytometry
☒	☐	MRI-based neuroimaging

Antibodies

Antibodies used	Anti-Flag (F1804, Sigma); IgG (ab171870, Abcam); Antibody dilution used for each antibody was 1: 100
Validation	Both the Anti-FLAG and IgG antibody as described above were previously used (Reference 17; Wang et al., 2014) for ChIP assays involving the same transgenic line 35S-PHR2-FLAG as used in this study.

ChIP-seq

Data deposition

☒ Confirm that both raw and final processed data have been deposited in a public database such as GEO.

☒ Confirm that you have deposited or provided access to graph files (e.g. BED files) for the called peaks.

Data access links<br>*May remain private before publication.*	ChIP-Seq data is submitted to BioProject PRJNA735744:<br>https://Dataview.ncbi.nlm.nih.gov/object/PRJNA735744?reviewer=hjvlce636jnenf75q90rcce5a0
Files in database submission	ChIP-Seq raw data has been submitted to NCBI SRA and contains 4 fastq files (2 biological replicates of FLAG-IP and INPUT each).
Genome browser session<br>(e.g. UCSC)	NOT USED

Methodology

Replicates	Each biological replicate was collected from a different replicate experiment.
Sequencing depth	150bp paired-end reads were collected for each sample. In total, around 53 million reads were obtained for each sample out of which 37 million reads uniquely mapped to rice reference genome in BWA-MEM2.
Antibodies	Anti-FLAG (F1804, Sigma)
Peak calling parameters	MACS2 was used for peak calling
Data quality	Data quality assessment process has been mentioned in the METHODS section in detail
Software	TRIM-Galore 0.6.4, BWA-MEM2, samtools-1.14, bedtools 2.29.2, deepTools 2.0, MACS2, IGV 2.8.9 (ChIP-Seq)
